# The Genomic Landscape of Compensatory Evolution

**DOI:** 10.1371/journal.pbio.1001935

**Published:** 2014-08-26

**Authors:** Béla Szamecz, Gábor Boross, Dorottya Kalapis, Károly Kovács, Gergely Fekete, Zoltán Farkas, Viktória Lázár, Mónika Hrtyan, Patrick Kemmeren, Marian J. A. Groot Koerkamp, Edit Rutkai, Frank C. P. Holstege, Balázs Papp, Csaba Pál

**Affiliations:** 1 Synthetic and Systems Biology Unit, Institute of Biochemistry, Biological Research Center, Szeged, Hungary; 2 Molecular Cancer Research, University Medical Center Utrecht, Utrecht, the Netherlands; 3 Institute for Biotechnology, Bay Zoltán Non-Profit Ltd., Szeged, Hungary; Institute of Science and Technology Austria (IST Austria), Austria

## Abstract

Adaptive evolution is generally assumed to progress through the accumulation of beneficial mutations. However, as deleterious mutations are common in natural populations, they generate a strong selection pressure to mitigate their detrimental effects through compensatory genetic changes. This process can potentially influence directions of adaptive evolution by enabling evolutionary routes that are otherwise inaccessible. Therefore, the extent to which compensatory mutations shape genomic evolution is of central importance. Here, we studied the capacity of the baker's yeast genome to compensate the complete loss of genes during evolution, and explored the long-term consequences of this process. We initiated laboratory evolutionary experiments with over 180 haploid baker's yeast genotypes, all of which initially displayed slow growth owing to the deletion of a single gene. Compensatory evolution following gene loss was rapid and pervasive: 68% of the genotypes reached near wild-type fitness through accumulation of adaptive mutations elsewhere in the genome. As compensatory mutations have associated fitness costs, genotypes with especially low fitnesses were more likely to be subjects of compensatory evolution. Genomic analysis revealed that as compensatory mutations were generally specific to the functional defect incurred, convergent evolution at the molecular level was extremely rare. Moreover, the majority of the gene expression changes due to gene deletion remained unrestored. Accordingly, compensatory evolution promoted genomic divergence of parallel evolving populations. However, these different evolutionary outcomes are not phenotypically equivalent, as they generated diverse growth phenotypes across environments. Taken together, these results indicate that gene loss initiates adaptive genomic changes that rapidly restores fitness, but this process has substantial pleiotropic effects on cellular physiology and evolvability upon environmental change. Our work also implies that gene content variation across species could be partly due to the action of compensatory evolution rather than the passive loss of genes.

## Abstract 2

The Genomic Landscape of Compensatory Evolution Laboratory selection experiment explains how organisms compensate for the loss of genes during evolution, and reveals the deleterious side-effects of this process when adapting to novel environments.

## Author Summary

While core cellular processes are generally conserved during evolution, the constituent genes differ somewhat between related species with similar lifestyles. Why should this be so? In this work, we propose that gene loss may initially be deleterious, but organisms can recover fitness by the accumulation of compensatory mutations elsewhere in the genome. To investigate this process in the laboratory, we investigated 180 haploid yeast strains, each of which initially displayed slow growth owing to the deletion of a single gene. Laboratory evolutionary experiments revealed that defects in a broad range of molecular processes can readily be compensated during evolution. Genomic analyses and functional assays demonstrated that compensatory evolution generates hidden genetic and physiological variation across parallel evolving lines, which can be revealed when the environment changes. Strikingly, despite nearly full recovery of fitness, the wild-type genomic expression pattern is generally not restored. Based on these results, we argue that genomes undergo major changes not simply to adapt to external conditions but also to compensate for previously accumulated deleterious mutations.

## Introduction

Deleterious, but non-lethal mutations are constantly generated and can hitchhike with adaptive mutations [1]. Consequently, such deleterious alleles are widespread in eukaryotic populations [2],[3]. For example, as high as 12% of the coding SNPs in yeast populations are deleterious [2]. Many of the observed functional variation in this species yield proteins with compromised or no activities [2], or lead to complete loss of genes with significant contribution to fitness (Text S1). Deleterious loss-of-function variants may occasionally revert to wild type, eventually perish from the population, or become compensated by mutations elsewhere in the genome. The third possibility, termed compensatory evolution, is the focus of our study. Theoretical works suggest that mutant subpopulations can cross fitness valleys by the simultaneous fixation of a compensatory mutation in the population [4],[5]. This process can also work in large populations and is facilitated by linkage of the two alleles [5].

Compensatory evolution appears to be common at many levels of molecular interactions. It is involved in the maintenance of RNA and protein secondary structures, it mitigates the costs of antibiotic resistance [6],[7], and allows rapid fitness recovery in populations with accumulated deleterious mutation loads [7]–[9]. Compensatory regulatory mutations also act to stabilize gene expression levels across species [10],[11], and conserve DNA-encoded nucleosome organization [12]. The most detailed experimental analyses on compensatory mutations for fixed deleterious mutations were performed in DNA bacteriophages [8],[13]–[15], bacteria [16],[17], and *Caenorhabditis elegans* [7],[9]. Three major patterns emerged from these studies. As the target size for compensatory mutations is typically much larger than that for reversion, compensation is more likely than reversion of deleterious mutations [13]. The rate of compensatory evolution increased with the severity of the deleterious fitness effects, and was not limited to functionally interacting partners of the mutated gene [15].

As regards the potential pleiotropic effects of compensatory evolution, our knowledge is rather limited, not least because it demands detailed exploration of the underlying molecular mechanisms of compensation. Compensatory mutations may enhance fitness either by reducing the need for the gene with the compromised function, or by restoring the efficiency of the affected molecular function [18]. For compensation of fitness costs of antibiotic resistance conferring mutations, restoration of function was the most common mechanism [18], but in other systems the relative importance of functional substitution and restoration is unknown. In the case of functional restoration (e.g., by enhanced dosage of a redundant duplicate of the disrupted gene), one might expect limited pleiotropic fitness effects of compensatory mutations across environmental conditions.

**Figure 1:**
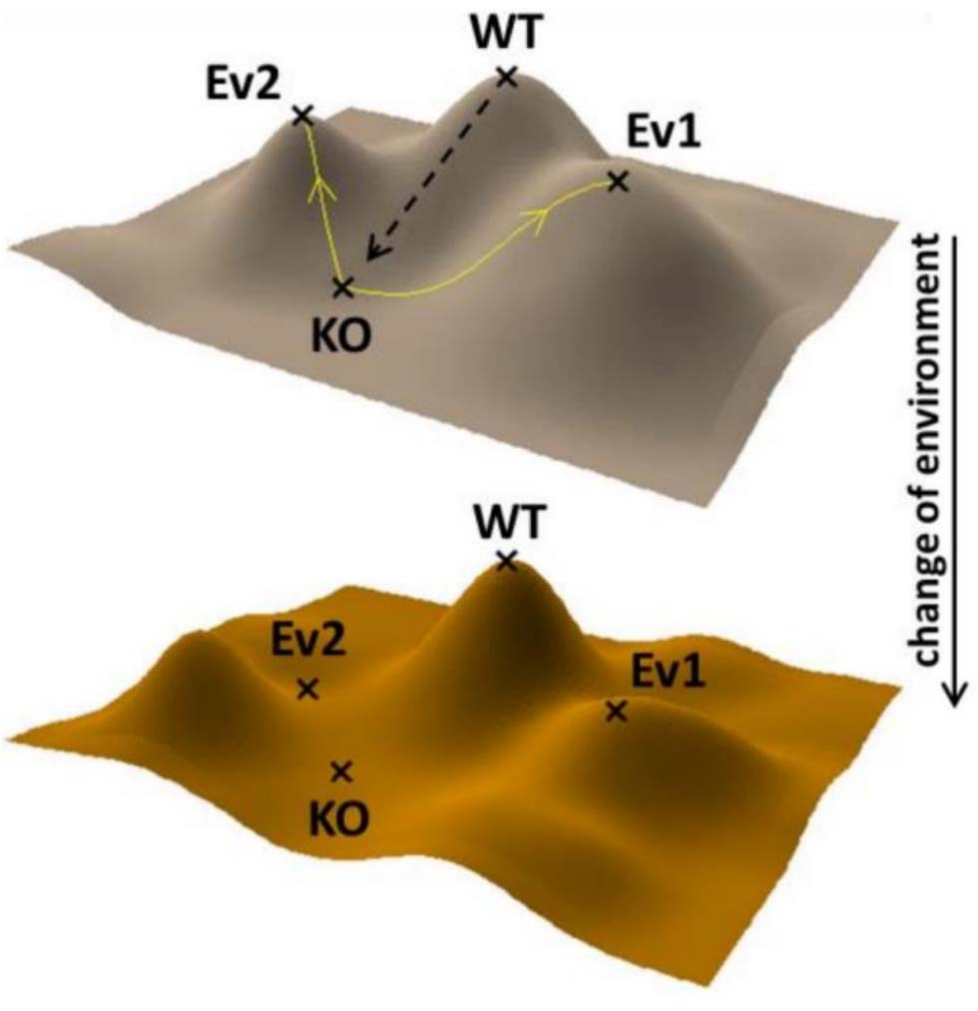
Compensatory evolution on the adaptive landscape. Schematic representation of the impact of compensatory evolution on the fitness landscape. The x and y axes on the landscape locate the network of neighboring genotypic states, while the z axis defines fitness in a single environment. Gene loss leads to a fitness valley (from WT to KO), while compensatory evolution can drive the population to different adaptive peaks (Ev1 versus Ev2). The upper fitness landscape shows the environment where compensatory evolution took place. The dashed arrow represents the original gene deletion event. Yellow lines represent different evolutionary routes. WT, wild type; KO, ancestor strain with a gene deletion.

Compensatory evolution following gene loss is of special interest [17]. Gene loss may be initiated by genetic drift and/or selection through antagonistic pleiotropy [17],[19]. As reversion to the wild-type state is less likely, gene loss may promote genetic changes that drive the populations to new adaptive peaks (Figure 1). It's reasonable to assume that compensatory mutations are generally specific to the gene defect, and multiple molecular mechanisms can restore fitness. Therefore, independently evolving populations carrying an inactivated gene are expected to diverge from each other. Moreover, if compensation mainly proceeds by reducing the need for the disrupted molecular function then compensatory evolution could have a large impact on cellular physiology and survival upon environmental change. Accordingly, the beneficial effects of compensatory mutations may frequently be conditional, and subsequent changes to the environment can reveal the hidden genetic variation across populations (Figure 1). The goal of the current study was to test this hypothesis by an integrated systems biology approach. Specifically, we aimed to determine the potential of the *Saccharomyces cerevisiae* genome to compensate for gene loss through compensatory evolution and to explore the long-term consequences of this process.

## Results

### Rapid Compensatory Evolution Following Gene Loss Is Common

**Figure 2:**
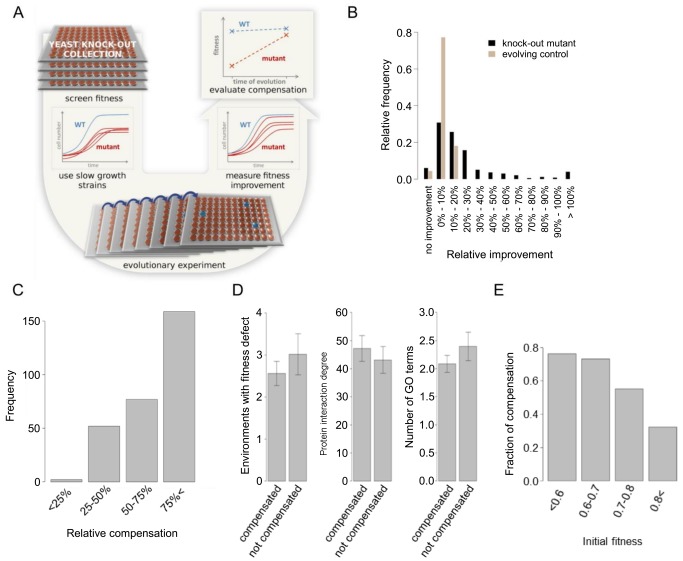
Compensation of fitness loss during laboratory evolution. (A) Experimental scheme to estimate evolutionary compensation of gene defects. See text for details. (B) Distribution of relative fitness improvement (RFI) of the knock-out mutant strains and the evolving control lineages (Table S1), where RFI = (evolved fitness/initial fitness)−1. (C) Relative compensation (RC) of the compensated knock-out mutant strains (Table S1), where RC is the fraction of the initial fitness defect that was compensated for during laboratory evolution (see Materials and Methods). (D) Compensation does not depend on pleiotropy (Table S1). The bars indicate mean ± standard error, Wilcoxon rank sum test *p*-values for the three comparisons are: 0.71, 0.44, and 0.36, respectively. (E) Genotypes with lower initial fitness were more likely to be compensated for during laboratory evolution (Table S1). Lines were divided into groups by initial fitness, the fraction of compensated lines among all the lines in the group is shown as bars (chi-squared test for trend in proportions, *p*<10^−13^, number of lines in the groups from left to right: 38, 56, 201, 337).

We initiated laboratory evolutionary experiments with 187 haploid single gene knock-out mutant strains, all of which initially showed slow (but non-zero) growth compared to the wild-type control in a standard laboratory medium (Figure 2A, for selection criteria, see Materials and Methods). These genes cover a wide range of molecular processes and functions (Table S1). Populations were cultivated in parallel (four replicate populations for each null mutation), resulting in 748 independently evolving lines. 0.5% of each culture was diluted into fresh medium every 48 hours, and populations were propagated for approximately 400 generations. To control for potential adaptation unrelated to compensatory evolution, we also established 22 populations starting from the isogenic wild-type genotype, referred to as evolving wild types. Next, all starting and evolved populations were subjected to high-throughput fitness measurements by monitoring growth rates in liquid cultures.

Fitness may increase during the course of laboratory evolution as a result of general adaptation to the environment and/or accumulation of compensatory mutations that suppress the deleterious effects of gene inactivation. Under the assumption that compensatory evolution was the dominant force in our experiments, fitness should not increase by the same extent in all lineages: genotypes that carry deleterious null mutations are further away from the optimal state and are hence expected to show large fitness gains (Figure 2A); this was indeed so. On average, the evolving wild-type control populations showed a small, but significant 5% fitness improvement. By contrast, the fitness of populations carrying a deleterious null mutation improved by 23% on average (Figure 2B), and many of them approximated wild-type fitness (Figure 2C; Table S1). On the basis of fitness measurements at multiple time points during laboratory evolution (see Methods), we also report that individual fitness trajectories often showed a saturating trend during the course of laboratory evolution (Figure S1).

The difference in fitness improvement is not due to the elevated mutation rate of mutant genotypes for two reasons. First, a previous study conducted a genome-wide screen with the aim to identify genes in *S. cerevisiae* that influence the rate of mutations [20]. While a large number of such genes have been found, only four of them were present in our gene set (*Δrad54*, *Δrad52*, *Δmre11*, and *Δrad50*). Second, fitness improvements of the corresponding single gene knock-out strains did not differ from the rest of the dataset (one-tailed Wilcoxon rank sum test, *p* = 0.89).

As previously [16], we defined compensatory evolution as a fitness increase that is disproportionally large relative to that in the evolving wild-type lines. Using this definition, 68% of the genotypes showed evidence of compensatory evolution (i.e., at least one of the four independently evolving populations fulfilled the above criteria). The corresponding genes cover a wide range of molecular and cellular processes (Table S1).

### Impact of Gene Pleiotropy and Dispensability on the Propensity for Compensation

Next, we compared the fitness improvements between evolved lines founded from the same gene deletion genotype versus those founded from different genotypes. This analysis revealed that not all genes were equally likely to be compensated as fitness gain differed significantly across genotypes (ANOVA, F(186) = 3.9, *p*<10^−14^) (see also Figure S2). It has been previously suggested that as mutations with especially large fitness effects tend to disrupt a broader range of molecular processes [21], such mutations may influence the number of mutational targets where compensatory evolution can occur [13]. We compiled three datasets that estimate different aspects of gene pleiotropy [22], including fitness under diverse environmental conditions (environmental pleiotropy), the number of protein-protein interactions (network pleiotropy), and the number of biological processes associated with a gene (multifunctionality). The extent of evolutionary compensation did not depend on any of the above mentioned features (Figure 2D). However, consistent with results of prior small-scale bacterial and viral evolutionary studies [13],[16], null mutations with more severe defects were more likely to be compensated (Figure 2E). This pattern probably reflects that the availability of compensatory mutations across the genome strongly depends on the fitness effect of the deleted gene. We provide a simple explanation of this phenomenon in the Discussion.

### Compensatory Evolution Promotes Genomic Diversification

**Figure 3:**
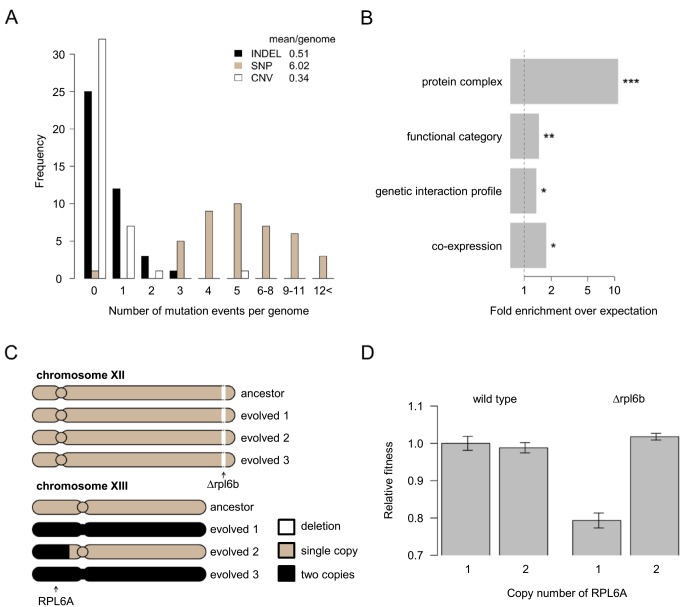
Genomic analyses of evolutionary compensation. (A) Distribution of different mutational events (Table S2). The inlet shows the color coding and the average value of total mutational events per genotype. (B) The originally deleted gene and the gene with identified *de novo* mutation participated more often in the same protein complex, were more often assigned to the same functional category and showed significantly more similar genetic interaction and expression profile than expected by random shuffling of the knock-out gene–mutated gene network. Dashed line represents no enrichment; */**/*** indicates *p*-value<0.05/0.01/0.001, respectively. The x axis is logarithmically scaled. (C) Δ*rpl6b* evolved lines showed duplication of the chromosomal region (or the complete chromosome) carrying a duplicate with redundant function (*RPL6A*). The gene positions are marked by arrows below the corresponding chromosome, copy numbers are shown by color codes. (D) Dosage compensation of Δrpl6b by increased copy number of RPL6A (Table S5). Copy number of *RPL6A* was increased by transforming the *RPL6A* bearing plasmid of the MoBY ORF Library. As the vector carries a selectable marker and a yeast centromere, the plasmid is present in one to three copies per cell. As a control, strains were transformed with the empty centromeric plasmid. Relative fitness was measured as colony sizes on agar plates, values were normalized to the wild-type control with a single genomic copy of RPL6A. All strains were grown on synthetic complete medium without uracil to select for the plasmids. Error bars show standard error.

To investigate the genomic changes underlying compensatory evolution, we re-sequenced the complete genomes of 41 independently evolved lines and the 14 corresponding ancestors, all of which showed large fitness improvements (Table S1). We focused on *de novo* mutations that accumulated during the course of laboratory evolution. Large-scale duplications (including segmental or whole chromosome duplication) were observed in 22% of the laboratory evolved lines. On average, six point mutations and 0.5 small insertions or deletions per clone were detected (Figure 3A; Table S2). The ratio of non-synonymous to synonymous mutations was significantly higher than expected by chance (*p* = 0.003, see Materials and Methods), indicating that the accumulation of these mutations was driven by adaptive evolution. On average, pairs of evolutionary lines founded from the same genotype shared 5.3% of their mutated genes, while the same figure was 0.1% for lines founded from different genotypes (Table S2). This result is in contrast to results of a prior bacterial study [23], where a strong signature of parallel evolution emerged at the gene level across parallel evolving laboratory populations. Despite the rarity of parallel evolution at the molecular level, a major unifying trend emerged: evolution preferentially affected genes that are functionally related to that of the disrupted gene (Figure 3B). Moreover, when the null mutation affected a protein complex subunit, another subunit of the same complex was mutated 10 times more often than expected by chance (Figure 3B). Taken together, these results indicate that deletion of any single gene drives adaptive genetic changes specific to the functional defect incurred.

### Pre-Existing Genetic Redundancy Has No Major Impact on Compensatory Evolution

Although duplicated genes with partially overlapping function are frequent in the yeast genome, we found no evidence that genetic changes affecting a duplicate of the disrupted gene provide a general mechanism of compensation in our evolved lines. First, our dataset contains 128 genes showing evidence for compensation, and only 25% of these genes have a duplicate in the yeast genome (i.e., at least 30% amino acid similarity between the two copies). This figure is a gross overestimate, as it includes very distant duplicates that most likely diverged functionally (Materials and Methods). Second, the subset of genes with a gene duplicate were not more likely to be compensated during laboratory evolution than the rest of the dataset (Chi-squared test, *p* = 0.54). Third, genome sequence analysis of the evolved lines revealed only one clear example where evolution proceeded through increasing the dosage of a gene duplicate with redundant function of the deleted gene (Figure 3C). All three studied evolved lines of *Δrpl6b* showed an increased copy number of the left arm of Chromosome XIII (Figure 3C). *RPL6B* is a non-essential gene and encodes a ribosomal 60S subunit protein L6B. The duplicated genomic regions of *Δrpl6b* evolved lines carry *RPL6A*, a duplicate copy of *RPL6B*. The two genes share 94% amino acid identity, have highly overlapping functions, and deletion of both genes confer a synthetic lethal phenotype [24]. On the basis of these observations, we propose that doubling the copy number of *RPL6A* through segmental duplication could be partly responsible for the improved fitness in the evolved lines carrying the *RPL6B* deletion. The hypothesis was tested by increasing the copy number of *RPL6A* in wild-type and Δrpl6b genetic backgrounds, respectively. As expected, an enhanced copy number of *RPL6A* substantially improved the fitness of *Δrpl6b*, but not that of the wild type (Figure 3D).

### Compensatory Evolution Does Not Restore Wild-Type Genomic Expression State

**Figure 4:**
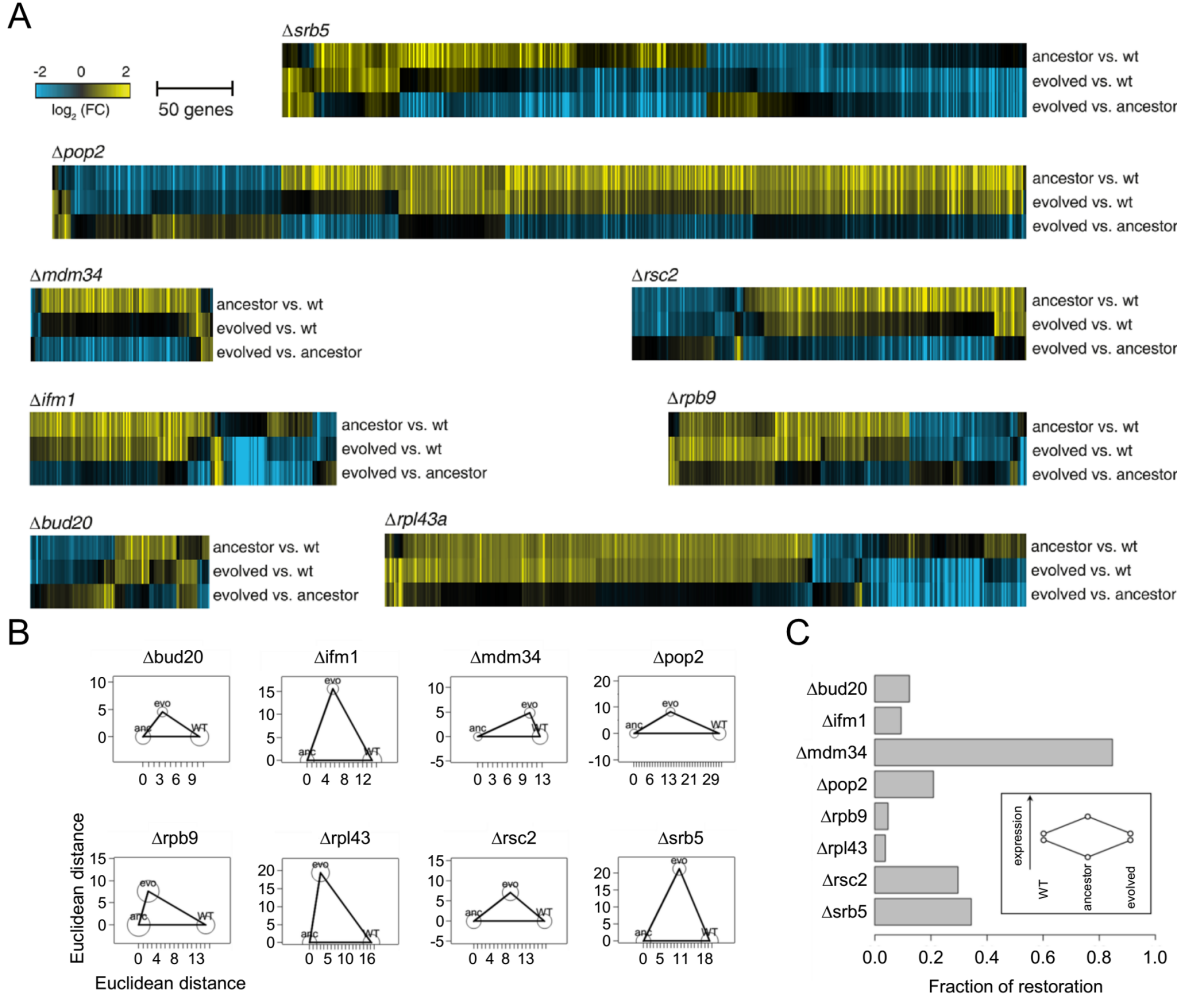
Comparisons of the transcriptome profiles of wild-type, ancestor, and evolved lines. (A) Heatmaps of transcriptome profiles of deletion mutants *Δrpl43a*, *Δpop2*, *Δmdm34*, *Δrsc2*, *Δifm1*, *Δrpb9*, and *Δbud20* and their corresponding evolved lines. For each deletion mutant, the fold-changes (FC) are shown for the ancestor strain versus the wild type, the evolved strain versus the wild type and the evolved strain versus the ancestor strain (Table S3). Color scales as indicated. Individual transcripts are depicted if they change significantly (FC>1.7, *p*<0.05) at least once in one of these comparisons. (B) The Euclidean distances of microarray profiles of the evolved evolutionary line from its ancestor and from wild type (WT) were calculated and normalized to the ancestor–wild type distance for each genotype. The distances of the points in the figure are proportional to the calculated profile distances. For each genotype triplet, distances were calculated on the basis of those genes that are differentially expressed in at least one of the pairwise comparisons. For each deletion strain, the edges of the triangle represent Euclidean distances of log_2_ mRNA expression fold-changes between the wild-type (WT), ancestor (anc), and evolved (evo) lines. To calculate these distances we used the average of four replicate expression measurements (two biological and two technical replicates). Circles around average values represent the Euclidean distance between the two biological replicates (calculated as the average based on the two technical replicates). For each genotype triplet, distances were calculated on the basis of those genes that are differentially expressed (FC>1.7, *p*<0.05) in at least one of the pairwise comparisons (Table S6). (C) Within the subset of genes that showed expression change upon gene deletion, the barplot shows the fraction of these genes that changed expression during evolution in the opposite direction (i.e., evolution towards restoration of wild-type expression level; see inset). With one major exception (lines disrupted in *mdm34*), only a small fraction of the expression changes were restored in the evolved lines (Table S6). The threshold for expression change was 1.7-fold-change and *p*<0.05, as in [62].

Compensatory evolution may restore wild-type physiology or generate novel alterations with respect to prior physiological states [25]. To investigate the relative contribution of these processes, eight genotypes carrying a deleterious gene deletion and one corresponding evolved line were selected for transcriptome analysis (see Materials and Methods for selection criteria). Using DNA microarrays, the global gene expression states were compared between the wild-type, the ancestral line, and the evolved lines carrying the same gene deletion (Figure 4A and 4B). As expected from prior studies [26], inactivation of genes with high fitness contribution altered the expression of a large number of genes across the genome (ranging between 81 to 588) (see Table S3). Next, the transcriptomic profiles were compared by calculating all pairwise combinations of Euclidean distances. The wild-type, the ancestral line, and the corresponding evolved lines generally showed substantial differences in their transcriptome profiles (Figure 4B), indicating that compensatory evolution drives the cell towards novel genomic expression states. Importantly, transcriptome profile distances between different genotypes was always higher than distances between replicate measurements of the same genotype (Figure 4B), implying that the substantial differences observed between evolved lines and wild-type cannot be attributed to measurement noise. As a further support, typically only 10%–30% of the genes with altered expression in the ancestral lines showed significant shift towards the wild-type expression level in the corresponding evolved lines (Figure 4C). Hence, despite substantial fitness improvements (>75% for all cases investigated), the majority of the gene expression changes due to gene deletion remained unrestored during evolution. These patterns were not attributable to growth rate regulated gene expression or copy number variation in the evolved lines (Figure S3).

### Compensatory Evolution Generates Diverse Growth Phenotypes across Environments

**Figure 5:**
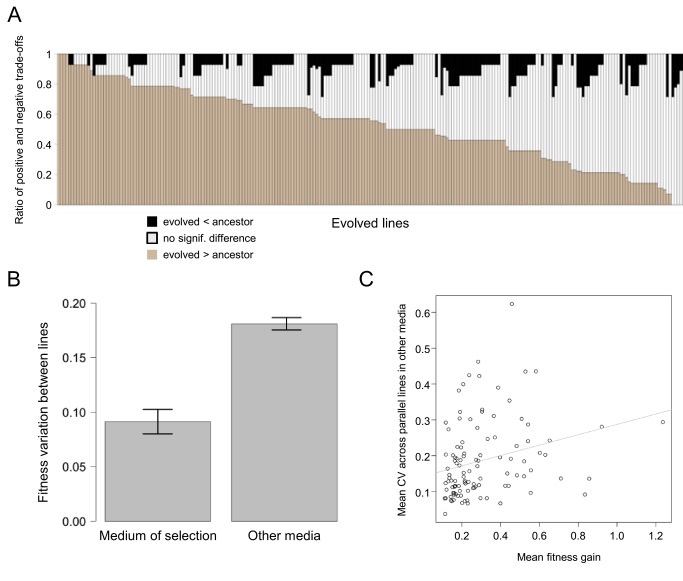
Large-scale phenotypic screen of evolved lines. (A) Fitness trade-offs in evolved lines carrying a deletion across 14 environments (Table S1). Lines are ranked according to the number of environments in which they display improved fitness (brown). Grey and black dots indicate conditions where the fitness of the line is statistically equal or lower, respectively, than that of the corresponding ancestor. (B) Fitness variation in independently evolving lines carrying the same gene deletion. The figure shows the coefficient of variation in the in the medium of selection (YPD) versus all other media (Table S1). The difference is highly significant (Wilcoxon rank sum test *p*-value<10^−7^). The bars indicate mean of the coefficients of variations ± standard error. (C) Gene deletions showing larger fitness gains have higher variance of fitness between replicate lines across other environments (Spearman rank correlation, rho = 0.36, *p* = 0.0001). Each point represents a gene deletion genotype. The x-axis shows the mean of the fitness gains of the parallel evolving replicates of a given gene deletion, while the y-axis shows the mean of the coefficient of variations measured in each alternative media between the parallel evolving replicates after 104 days of lab evolution (Table S1). The gray line indicates fit by linear regression.

Taken together, compensatory evolution following gene loss did not restore wild-type genomic expression and promoted genomic divergence across populations. Are these evolutionary outcomes phenotypically completely equivalent? This problem was first addressed by monitoring the fitness of 237 evolved populations in 14 environmental settings, including previously tested nutrients and stress factors [27]. Prior to evolution, genotypes carrying a gene deletion generally displayed slow growth in most environments (Table S1). The situation was far more complex following laboratory evolution. Considering all possible pairs of population-environment combinations, fitness improved in 52%, and declined in 8% of the cases (Figure 5A). Moreover, independently evolved populations carrying the same disrupted gene showed more fitness variation across the 14 tested conditions than in the environment they had been exposed to during laboratory evolution (Figure 5B, *p*<10^−7^), while evolved wild-type populations did not show such a difference (*p* = 0.93, coefficient of variations compared by Z-test). Furthermore, the degree of fitness variation across conditions was especially high for gene deletions that showed large fitness gains during compensatory evolution (Spearman rho = 0.36, *p* = 10^−4^) (Figure 5C). These results indicate that the level of discernible heterogeneity in fitness was relatively low in the evolved populations founded from the same genotype, but the variation can be uncovered upon environmental change.

Finally, our analysis revealed a few instances where the laboratory evolved lines displayed significantly higher than wild-type fitness in specific environments (Table S1). Most notably, the evolved *Δrpl6b* and *Δatp11* lines displayed 24%–26% fitness increase compared to that of the wild type in a medium containing sodium chloride (Table S1), a result that was confirmed by additional independent colony size assays with high replicate number (*n* = 20, Wilcoxon rank-sum test *p*<10^−4^ in all cases). Moreover, the fitnesses of these lines in this medium surpassed all that of the 22 evolved wild-type controls. These results are all the more remarkable, as the corresponding ancestral *Δrpl6b* and *Δatp11* strains showed fitness values significantly lower than wild type under all environmental conditions considered. These preliminary results indicate that gene loss can promote adaptive evolution towards novel environments, a possibility that will be explored further in a future work.

### A Case Study Reveals the Fitness Cost and Condition Dependence of Compensatory Evolution

**Figure 6:**
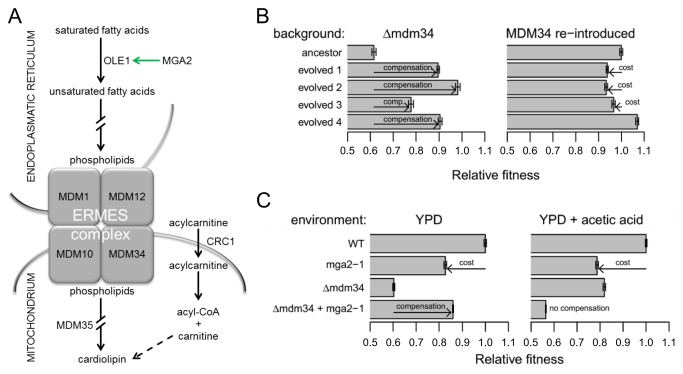
Compensation of the *MDM34* gene deletion. (A) The cardiolipin synthesis pathway with an emphasis on the ERMES complex. The complex tethers the endoplasmatic reticulum to the mitochondria, and is central for the transfer of phospholipids between the two compartments. *De novo* mutations in the independent evolutionary lines affected different, but related cellular subsystems, including upregulation of the unsaturated fatty acid synthesis (*MGA2*), another step of the cardiolipin synthesis pathway downstream of the ERMES complex (*MDM35*), and another mitochondrial transport process (*CRC1*), which most likely affects respiration by modulating the interaction between carnitine and cardiolipin. For further details on the underlying mechanisms see Text S1. The green arrow represents transcriptional upregulation; the dashed arrow indicates indirect positive effect. The mutations in *MGA2*, *MDM35*, and *CRC1* genes were found in Δ*mdm34* evolved lines 1, 3, and 4, respectively. (B) The cumulative fitness effects of the compensatory mutations in Δ*mdm34* and “wild type” (Δ*mdm34*+*MDM34* reintroduced) backgrounds (Table S7). (C) Epistatic interactions between mutations in two environments (Table S7). The bars in (B) and (C) indicate means ± standard error. Arrows indicate fitness costs and the extent of compensation.

Next, we conducted an in-depth genetic analysis with the *MDM34* deletion with the aim of deciphering the molecular mechanisms and/or potential fitness costs of compensatory mutations (Text S1). This gene codes for a component of the ERMES protein complex, and is involved in the exchange of phospholipids between mitochondria and the endoplasmatic reticulum (Figure 6A). Disruption of this gene yields impaired cardiolipin synthesis [28], as an insufficient amount of unsaturated fatty acids reaches the mitochondria (Figure 6A). Laboratory-evolved lines carrying deletion in this gene substantially improved fitness in the medium of selection (Table S1), but the putative cellular mechanisms of compensation were remarkably different across populations (Figures 6A and S4). The native copy of *MDM34* was reinserted into the ancestral line and four evolved lines carrying the same deletion (Δ*mdm34*). The analysis revealed that the net effect of mutations in three evolved lines were deleterious in the presence of *MDM34* (Figure 6B). Next, we concentrated on a specific mutation observed in *MGA2*, a gene involved in the regulation of unsaturated fatty acid biosynthesis (Figure 6A; Text S1). Inserting the observed mutations (*mga2-1*) into wild type and Δ*mdm34* resulted in very similar conclusions. *mga2-1* and Δ*mdm34* showed strong sign-epistasis [29]: they were independently deleterious but significantly less so when they occurred together (Figure 6C). Moreover, the capacity of *mga2-1* to compensate the loss of *MDM34* was restricted to non-acidic conditions (Figure 6C), probably because of the misregulation of the corresponding stress-induced pathway under low pH (Text S1).

### Evolutionary Compensation by Loss-of-Function Mutation

Our dataset contains 21 independent point mutations that occurred during laboratory evolution and generated in-frame stop codons. Most notably, a mutation in *WHI2* emerged in an evolving *Δrpb9* line, which shortened the coding region from 480 to 133 codons, and hence most likely resulted in a non-functional protein.

**Figure 7:**
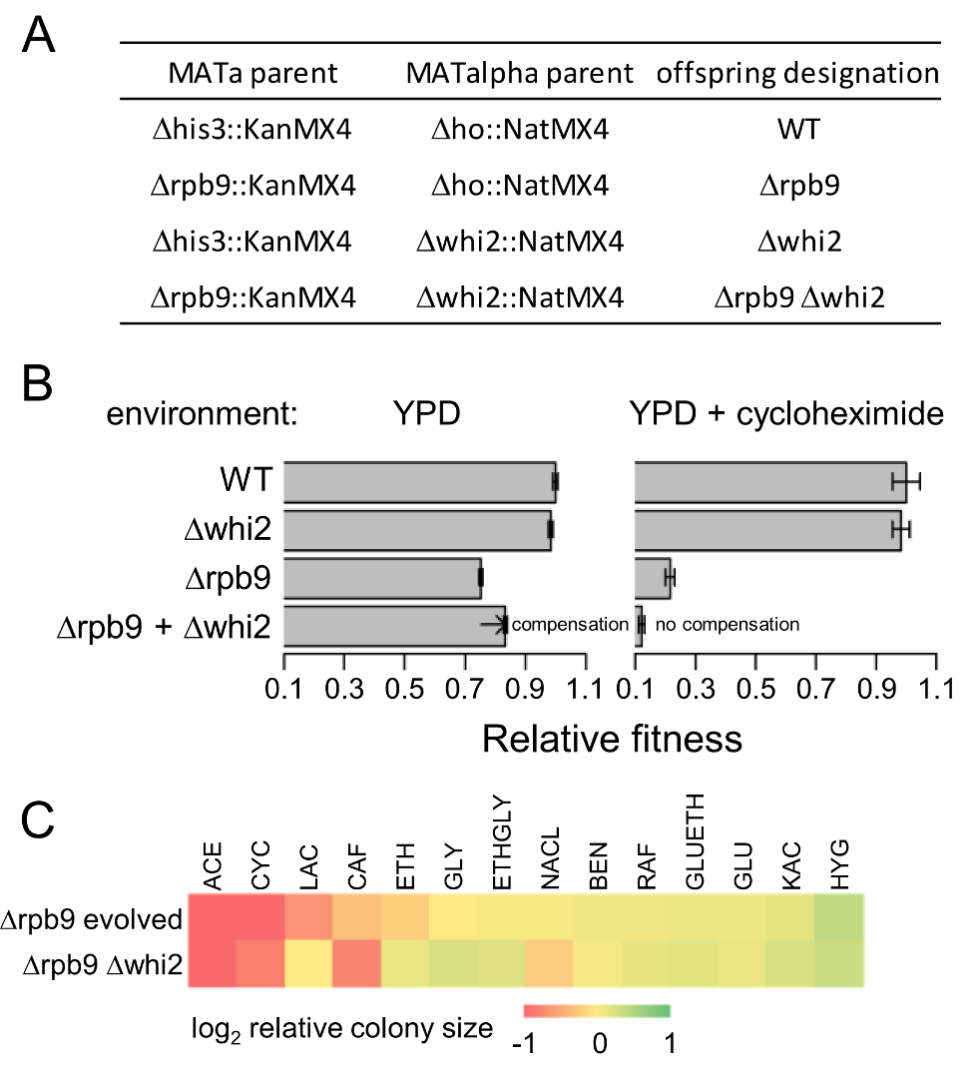
Environment-dependent compensation by a loss-of-function mutation. (A) *Δrpb9* and *Δwhi2* mutations were crossed by SGA using haploid parental strains as shown. To compare the double mutant *Δrpb9 Δwhi2* with the wild-type control and corresponding single mutants, the resistance cassettes required by the SGA method were introduced into wild-type and single mutants by crossing them with parental strains where the corresponding resistance cassettes reside at a non-functional locus (*Δhis3*::KanMX4 and *Δho*::NatMX4). (B) Relative fitness was measured as colony sizes on YPD and YPD supplemented with cycloheximide (CYC), values were normalized to WT. The arrow shows the extent of compensation of *Δrpb9* by *Δwhi2* on glucose medium (Wilcoxon rank sum test *p* = 0.005, error bars show standard error) (Table S8). (C) Relative fitness of *Δrpb9* replicate evolving line 2 and *Δrpb9 Δwhi2* double mutant were measured as colony sizes grown on different media. Genotypes are indicated on the left, the growth media are indicated above the heat map. For media composition and abbreviations, see Table S4. Values are normalized to *Δrpb9* ancestor. Log_2_ values are shown according to the color coding (Table S8).

To test the impact of loss of *WHI2* function on fitness and compensation, *Δwhi2* was introduced into *Δrpb9* cells using synthetic genetic array methodology (Figure 7A and 7B) [30]. In agreement with expectation, deletion of *WHI2* partly suppressed the harmful effect of the *RPB9* deletion (Figure 7B). *RPB9* is an RNA polymerase II subunit, and its deletion leads to elevated transcriptional error rate [31] and in turn, to proteotoxic stress [32], which can result in cell cycle arrest [33]. *WHI2* is known to be required for general stress response [34] and cell cycle arrest [35]. We speculate that less stringent cell cycle control due to *WHI2* deletion is favorable in *Δrpb9* (see also [36]).

Next, the fitness impact of *WHI2* deletion was evaluated across 14 environments. The fitnesses of the *Δrpb9 Δwhi2* strain varied strongly across conditions, and showed correlation with that of the evolved *Δrpb9* line, which carried the *WHI2* non-sense mutations (Spearman rho = 0.77, *p*<0.005) (see Figure 7C). Most notably, the compensation of *Δrpb9* by *Δwhi2* was completely abolished in the presence of cycloheximide (Figure 7B). We conclude that the compensatory effect of *WHI2* deletion is plastic across environments.

## Discussion

Our work addresses one of the most long-standing debates in evolution. Since the early 1920s, Ronald Fisher pioneered the view that adaptation is by and large a hill climbing process: it proceeds through progressive accumulation of beneficial mutations [37],[38]. However, as slightly deleterious mutations are far more abundant, they have a significant contribution to genetic variation in natural populations [2]. In the long run, the wealth of such detrimental mutations is expected to promote fixation of compensatory mutations elsewhere in the genome. This work focused on a specific aspect of this problem, and asked whether deleterious gene loss events promote adaptive genetic changes and what the side consequences of such a process might be. To systematically study compensatory evolution following gene loss, we initiated laboratory evolutionary experiments with over 180 haploid yeast genotypes, all of which initially displayed slow growth owing to the deletion of a single gene, and investigated the genomic and phenotypic capacities of the evolved lines in detail. Thanks to the exceptionally large-scale analysis of our study, the following major conclusions can be drawn.

**Figure 8:**
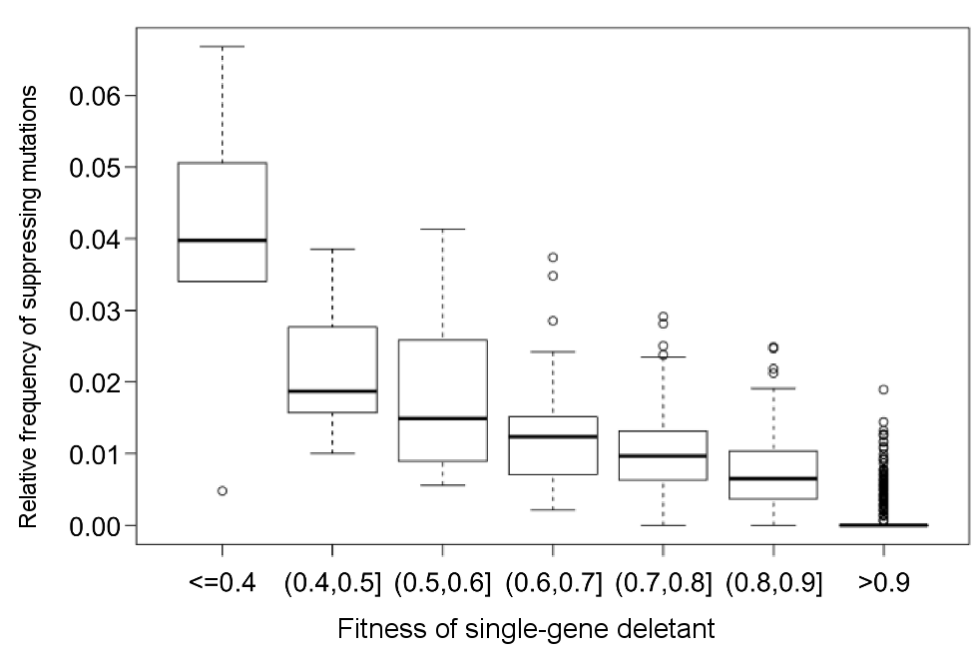
Strongly deleterious gene deletions can be suppressed by a large number of other null mutations according to a genome-wide genetic interaction study. The plot shows the relationship between the fitness of a given single-gene deletion strain and the fraction of other genes across the genome whose deletion suppresses the fitness effect of this mutation (Table S9). Boxplots present the median and first and third quartiles, with whiskers showing either the maximum (minimum) value or 1.5 times the interquartile range of the data. Spearman correlation on raw data: rho = −0.69, *p*<10^−16^, *n* = 3880. We note that using the fraction of suppressive interactions among all genetic interactions displayed by a given gene yields a very similar result (rho = −0.69, *p*<10^−16^), indicating that the relationship is not simply due to the fact that slow-growing strains generally display especially large numbers of both positive and negative interactions [21]. Information on suppression genetic interactions and single-deletion fitness comes from a global genetic interaction map of yeast [21]. Suppression interactions were defined as in previously [70]. In brief, deletion of gene *B* suppresses deletion of gene *A* if their fitness values obey the following rules: *F_A_*<*F_B_* and *F_AB_*>*F_A_*+*σ_A_* (where *F_A_*, *F_B_*, and *F_AB_* are the fitness measures of single deletants *A*, *B*, and the double deletant *AB*, respectively, and *σ_A_* is the standard deviation of *F_A_*). One important caveat is that as this simple analysis considers null mutations only, the results should be considered preliminary.

First, compensatory evolution following gene loss was pervasive: 68% of the deleterious, but non-lethal gene disruptions were compensated through the accumulation of adaptive mutations elsewhere in the genome (Figure 2B). Furthermore, in agreement with prior bacterial studies [16],[17], the process was strikingly rapid. As the set of disrupted genes are functionally very diverse (Table S1), it appears that defects in a broad range of molecular processes can readily be compensated during evolution.However, we and others [17] also found that not all genotypes are equally likely to be recovered during laboratory evolution. Therefore, future works should clarify the exact molecular, functional, and systems level gene properties that influence compensability. Second, our large-scale study indicates that the extent of fitness loss due to gene disruption is one if not the strongest predictor of compensatory evolution (Figure 2E). Although this relationship has been observed previously in small-scale studies [16], the reasons remained largely unknown. One may argue that the spread of compensatory mutations with mild beneficial effects would have taken many more than 400 generations to reach fixation [16]. Although this explanation cannot be excluded, there is another intriguing possibility [13]. Consistent with Fisher's geometric model [37],[38], fitness improvement in populations close to an optimal state can only be achieved by relatively rare mutations with small effects. However, when a population with a gene defect is further away from a fitness peak, compensatory evolution may proceed through a wider range of mutations, including ones that have deleterious side effects. Two lines of evidence are consistent with this scenario. Compensatory evolution has associated pleiotropic effects (Figures 5 and 6C). Moreover, the theory predicts that compensatory mutations should be especially frequent in the case of strongly deleterious null mutations. An analysis based on data of a prior genome-wide genetic interaction study [21] suggests that it may indeed be so (Figure 8).

Third, genomic analysis of the evolved lines revealed that deletion of any single gene drives adaptive genetic changes specific to the functional defect incurred (Figure 3B), and consequently convergent evolution at the molecular level was extremely rare. In agreement with a prior bacterial evolutionary study [17], we found that gene duplication has only a minor role during compensatory evolution following gene loss. A more general issue is the extent to which mutations that affect gene expression could alone recover fitness [17],[39]. Although genetic changes in putative promoter regions were not overrepresented in our dataset (Binomial test, *p* = 0.87), 21 observed point mutations generated in-frame stop codons, most likely yielding proteins with compromised or no activities (see also Figure 7). These results indicate that fitness recovery following gene loss can partly be achieved purely through inactivation of other genes.

Fourth, compensatory evolution promoted divergence of genomic diversification, and shifted the evolved population towards novel genomic expression states (Figure 4B). Despite substantial fitness improvements, the majority of the gene expression changes due to gene deletion remained unrestored during evolution. This finding is consistent with prior works arguing that no clear relationship exists between the change in mRNA expression of a gene and its requirement for growth in the same condition [40].

Fifth, independently evolved populations showed substantial fitness variation across environments that they had not been exposed to during laboratory evolution (Figure 5). These results suggest that accumulation of adaptive mutations during compensatory evolution generated substantial genetic differences between populations, and this variation can be uncovered upon environmental change.

Taken together, several lines of evidence indicate that fitness gains in the evolved lines reflect accumulation of gene specific compensatory mutations rather than a global adaptation: (i) evolving wild-type control populations showed only minor changes in fitness, (ii) the rate of adaptation was genotype specific, (ii) convergence at the molecular across genotypes was extremely rare, (iv) evolution preferentially affected genes that are functionally related to that of the disrupted gene, and (v) compensatory mutations had no beneficial impact in a wild-type genetic background.

The above results encouraged us to distinguish between two evolutionary scenarios. Organisms may attempt to restore the disrupted molecular function through mutations in genes with redundant functions (functional restoration). Alternatively, they may aim to minimize the cellular damage incurred by gene disruption (functional replacement). While the possibility of full functional restoration cannot be excluded, the rarity of compensation through mutations in gene duplicates and the plasticity of compensatory mutational effects across environments are consistent with the second scenario. Indeed, our work demonstrates that gene loss promotes genetic changes that have a large impact on evolutionary diversification, genomic expression, and viability upon environmental change. An important implication of our study is that the beneficial effects of compensatory mutations should frequently be conditional, and subsequent changes to the environment can reveal the hidden fitness effects (beneficial and detrimental alike). Lack of restoration of fitness across environments is broadly consistent with the emerging view that epistatic interactions are plastic across conditions [41],[42].

The perspective offered in this work leads to the re-formulation of several fundamental questions. First, it sheds light on an evolutionary paradox: while core cellular processes are generally conserved during evolution [43], the constituent genes are partly different across related species with similar lifestyles. We propose that gene content variation across species is partly due to the action of compensatory evolution and may not need to reflect changes in environmental conditions and the consequent passive loss of genes. Although the exact population genetic conditions facilitating this process remain to be elucidated, several observations are consistent with this view. Most notably, the phylogenetic conservation of indispensable genes depends on how easily the gene can be functionally replaced through enhanced expression of other genes [44]. Second, it has been suggested that deleterious mutations may act as stepping stones in adaptive evolution by providing access to fitness peaks that are not otherwise accessible [45],[46]. Indeed, our analysis revealed a few instances where the laboratory evolved lines displayed significantly higher than wild-type fitness in specific environments. Finally, given the prevalence of gene loss events during tumorigenesis, future work should elucidate whether similar processes drive the somatic evolution of cancer [47].

## Materials and Methods

### Yeast Strains and Media

All strains used in this study were derived from the BY4741 *S. cerevisiae* parental strain. Non-essential single-gene deletion strains from the haploid yeast deletion collection [40] (MATa; his3Δ 1; leu2Δ 0; met15Δ 0; ura3Δ 0; xxx::KanMX4) were used to systematically identify all gene disruptions with a significant growth defect. Slow-growing mutants were identified in two steps. An earlier study identified 671 gene deletants in diploid background, which showed a significant fitness defect on both rich and synthetic media [48]. We thus measured fitness of the corresponding MATa haploid strains by recording their growth curves in liquid media. We identified 187 deletants showing at least 10% growth rate defect, which constituted the set of ancestral strains subjected to laboratory evolution (for details of growth measurements see below).

The slow-growing yeast deletants used in this study are listed in Table S1. The evolutionary experiment was conducted using rich liquid medium (YPD, 1% yeast extract, 2% peptone, 2% glucose). Solid media were prepared using 2% agar, which were found to be optimal for reproducible colony size measurement. Details on the media used in the phenotypic profiling experiment can be found in Table S4. Oleic acid and stearic acid was dissolved in DMSO as a 100 mM stock and added to the medium after autoclaving to a final concentration of 0.1 mM.

### Laboratory Evolution

Compensatory adaptation refers to fitness gains in a gene deletion strain that are greater than fitness gains occurring in an isogenic wild-type strain. We conducted a series of laboratory evolutionary experiments using four independent populations of each of the 187 slow-growing deletants along with 22 independent lineages of an isogenic wild-type strain (referred to as *evolving* wild types). The *YOR202W* deletion strain was used as evolving wild-type control because the fitness of this strain is indistinguishable from the BY4741 parental wild-type strain [19]. Moreover, this strain carries the KanMX4 cassette in the nonfunctional *his3*Δ*1* allele, thus it was possible to control for the reported mutation-generating effect of the KanMX4 cassette [36]. All strains were inoculated into randomly selected positions of 96-well plates. Four wells in different positions were not inoculated by cells to help plate identification and orientation. Cells were grown in standard laboratory rich media to minimize selection pressure originating from nutrient limitation. The presence of the KanXM4 cassette was not selected for during the evolutionary experiment, since G418 was omitted from the medium for two reasons. First, using G418 at 200 mg/l concentration decreases the growth rate of the unevolved wild-type control strain (unpublished data) and might lead to selection for increased resistance. Second, the usage of the drug at a growth-limiting concentration may induce mutagenesis through environmental stress response. To provide optimal growth conditions, plates were covered with sandwich cover (Enzyscreeen BV), shaken at 350 rpm, and incubated at 30°C. Using a handheld replicator, ∼10^5^ cells (∼0.5 µl sample volume) were transferred every second day to 100 µl of fresh medium in 96-well plates resulting in ∼7.6 generations between transfers. The experiment was run for 104 days (∼400 generations total) and samples from days 0, 26, 52, 78, and 104 were frozen in 15% glycerol and kept at −80°C until fitness measurement. Cross-contamination events were regularly checked by PCR and visual inspection of empty wells (unpublished data).

### High-Throughput Fitness Measurements

We used established protocols specifically designed to measure fitness in yeast populations [49]. Growth was assayed by monitoring the optical density (OD_600_) of liquid cultures of each strain using 384-well microtiter plates containing YPD medium (as during the evolutionary experiments). We used relative growth rate as a proxy for relative fitness (see below). Compared to laborious competition based fitness assays, this protocol allows estimating growth rate on a relatively large scale in an environment that is nearly identical to the one used in the evolutionary experiments.

### Growth Curve Recording

Starter cultures were inoculated from frozen samples using 96-well plates. The starter plates were grown for 48 hours under identical conditions to the evolutionary experiment. 384-well plates filled with 60 µl rich medium per well were inoculated for growth curve recording from the starter plates using pintool with 1.58 mm floating pins. The pintool was moved by a Microlab Starlet liquid handling workstation (Hamilton Bonaduz AG) to provide uniform inoculum across all samples. The median blank corrected initial OD_600_ of the wells was 0.027. Each 384-well plate were inoculated with four different starter plates: one plate having the unevolved wild-type control as a reference strain in all wells in order to estimate various within-plate measurement biases, and three plates containing the same set of mutants from three of the five time points of the evolutionary experiment. The 384-well plates were incubated at 30°C in an STX44 (LiCONiC AG) automated incubator with alternating shaking speed every minute between 1,000 rpm and 1,200 rpm. Plates were transferred by a Microlab Swap 420 robotic arm (Hamilton Bonaduz AG) to Powerwave XS2 plate readers (BioTek Instruments Inc) every 20 minutes and cell growth was followed by recording the optical density at 600 nm. Six technical replicate measurements were executed on all strains sampled from each time-point of the evolutionary experiment. Measurements with growth curve irregularities were automatically removed. Only those strains were further analyzed where at least four technical replicate measurements remained after this quality control step.

### Growth Curve Analysis

Growth rate was calculated from the obtained growth curves following an established procedure [49],[50]. To eliminate potential within-plate effects that might cause measurement bias, growth rates were normalized by the growth rate of neighboring reference wells that contained the wild-type controls. For each strain and each evolutionary time point, relative fitness was calculated as the median of the normalized growth rates of the technical replicates divided by the median growth rate of the wild-type controls. At day 0, the technical replicate measurements of the isogenic independently evolving lines were combined to calculate median ancestral fitness since by that time these populations had no independent evolutionary history. Stringent criteria were used to define the set of ancestor strains with substantial growth rate defect: a minimum of 10% fitness drop was required compared to the wild-type controls; significance was determined by one-tailed Wilcoxon rank sum test, *p*-value was corrected with a false discovery rate of 0.05.

### Identifying Lines Showing a Significant Compensatory Adaptation

To determine whether the fitness defect of a given knock-out strain became compensated during the evolutionary experiment two criteria must have been met: First, the growth rate improvement had to be significant (one-tailed Wilcoxon rank sum test, *p*-value corrected with a false discovery rate of 0.05). Second, the growth rate increment of the knock-out strain had to be disproportionally larger than that of the evolving wild-type control strains. To test whether fitness gain in a knockout is higher than those occurring in the evolving control lines, we first fitted a normal distribution to the fitness improvement values of the evolving control lines. Next, we defined a fitness improvement cutoff, so that the probability that an evolving control line would show an improvement at least that high is less than 0.05.

To evaluate the extent of evolutionary compensation, a relative compensation index was calculated according to the following formula:
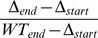
where WT and Δ means median normalized growth rate of the evolving wild-type control and the knock-out strain, respectively, measured before (start) and after (end) the evolutionary experiment. Thus, a relative compensation of 1 indicates that the knock-out strain reached the same fitness after evolution as the evolving wild-type control strains. See Table S1 for the whole dataset.

### Phenotypic Profiling across Environmental Conditions

To study the pleiotropic effects of compensatory adaptation, we measured the fitnesses of 237 evolved lines carrying a single gene deletion, all evolved wild-type control lines along with the corresponding ancestors across various environmental conditions. As this experiment demands high-throughput analyses (over 14,000 data points), fitness was estimated by colony size on solid agar media. Moreover, it allowed direct comparison of the reliability of our measurements to results of a previous study (Figure S5).

We prepared solid agar media of 14 different compositions to expose the strains to fundamentally diverse environments and to obtain sufficient throughput. Our list of 14 growth media was primarily based on a previous study [27] and included various carbon sources and stress conditions (Table S4). A robotized replicating system was set up for colony size based fitness measurement. The system consists of a Microlab Starlet liquid handling workstation (Hamilton Bonaduz AG) equipped with a pintool with 768 pins (S&P Robotics Inc) and a custom-made pintool sterilization station. Several aspects of the replication procedure had been experimentally customized to achieve uniform, reproducible inoculation of yeast cells.

Fitness of the ancestor (day 0) and evolved strains (day 104) was approximated by measuring colony sizes of ordered arrays of strains at 768 density. First, four different 96-well plates of the evolutionary experiment were scaled up to arrays of 384 colonies: one having the unevolved wild-type control in all positions, and three different plates of the mutant set from the same time point. Then pairs of 384 arrays with corresponding strains from day 0 and 104 were combined to reach 768 density. With this set up, all evolving replicate lines derived from the same ancestral genotype from both day 0 and day 104 were grown on the same 768 plate to exclude potential plate-to-plate variations when comparing colony growth of ancestor and evolved lines. Four technical replicates of these 768 arrays were transferred into each of the 14 different media.

After acclimatization to the media at 30°C for 48 hours the plates were replicated again onto the same type of media and photographed after 48 hours of incubation at 30°C. Digital images were processed to calculate colony sizes, and potential systematic biases in colony growth were eliminated (Text S1). For each growth environment, fitness of each original knock-out genotype at day zero and each independently evolving line at day 104 was determined as the median of the size of replicate colonies. The reliability of our experimental setup and data processing was confirmed by comparing the fitness measurements of ancestral knock-out strains with the published data of Dudley and colleagues (Figure S5) [27].

To determine whether an ancestor genotype shows a significantly altered fitness compared to the wild-type control in a given environment, we used a Wilcoxon rank sum test (with *p*-value corrected for each condition with a false discovery rate of 0.05). The same statistical test was used to determine whether the fitness of an evolved line is different from that of its ancestor in a given environment. See result in Table S1.

### Genome Sequencing

To reveal the underlying molecular mechanisms of compensation, we subjected 41 strains to whole-genome re-sequencing. Our list of sequenced strains primarily included genotypes with large initial fitness defect, substantial fitness improvement and gradual fitness increase over the course of evolution. To be able to detect parallel evolution at the molecular level, we selected two to four independently evolving lines of each ancestor genotype for sequencing. Overall, 41 evolved lines from 14 deletion strains were chosen along with their corresponding ancestor strains. Candidates were re-streaked and single clones were isolated and their fitness increase was confirmed by growth curve recording.

Genomic DNA was prepared using a glass bead lysis protocol: clones were inoculated into 5 ml YPD+G418 (200 mg/l) and grown to saturation at 30°C. Cells were pelleted and resuspended in 500 µl of lyis buffer (1% SDS, 50 mM EDTA, 100 mM Tris [pH 8]). Cells were mechanically disrupted by vortexing for 3 minutes at high speed with 500 µl glass bead (500 µm, acid washed). After adding 275 µl 7 M ammonium acetate, samples were incubated at 65°C for 5 minutes, followed by a second incubation on ice for 5 minutes. The samples were extracted with chloroform∶isoamyl alcohol (24∶1) and centrifuged for 10 minutes. The aqueous layer was transferred into a new tube and precipitated with 1 ml isopropanol, pelleted and washed with 70% ethanol, and resuspended in 500 µl RNaseA solution (50 ng/ml). After 30 minutes RNaseA treatment at room temperature, samples were chloroform∶isoamyl alcohol (24∶1) extracted, precipitated with 50 µl sodium acetate (3 M [pH 5.2]) and 1,250 µl ethanol, pelleted and washed with 70% ethanol. Finally, the genomic DNA was dissolved in water. The steps of re-sequencing was done by the UD-GenoMed Medical Genomic Technologies Ltd: amplified genomic shotgun libraries were run on the Illumina HighScan SC with 1×100 bp single read module resulting in an average coverage of about 80×. Reads were aligned to the *S. cerevisiae* EF4 genome assembly using the BWA software package [51] having the genomic repeats masked using RepeatMasking [52]. Variant calling was performed using the GATK software package [53]. Genomic single-nucleotide polymorphisms with less than 200 phred-scaled quality score or lower than 0.3 mutant/reference ratio were ignored. Duplications of large chromosomal segments or whole chromosomes were identified as increased read coverage of certain regions. Elevated read coverage of regions with a minimum of 25 kb length were accepted as duplications if both the Control-FREEC [54] (Wilcoxon rank-sum test, *p*<0.01) and the CNV-seq [55] (*p*<0.0001) software predicted significant alteration from the read coverage of the reference genome.

Our primary aim was to analyze *de novo* mutational events. *De novo* mutations were identified as alterations from the reference genome specifically found in the evolved lines but not present in the ancestral strains. Mutations, which occurred before our evolutionary experiment but after the gene knock-out, are referred to as secondary ancestor mutations. These mutations were identified in the ancestral strains as SNPs and indels present only in the corresponding ancestor strain, not in any other ancestral strains. The rationale behind this consideration is not to classify mutations accumulated in the parental strain of the mutant library prior to the generation of the knock-out strain as a secondary ancestor mutation. The list of identified mutations can be found in Table S2.

### Ratio of Non-Synonymous to Synonymous SNPs

Whole-genome re-sequencing revealed that 86% of SNPs in the coding regions were non-synonymous. To statistically test whether the ratio of non-synonymous to synonymous SNPs was higher than expected based on a neutral model of evolution, we employed the method of Barrick and colleagues [56]. Briefly, we took all different point mutations observed in protein coding regions and calculated the probability that 86% or more substitutions would result in a non-synonymous substitution if it occurred in a random coding position. The excess of non-synonymous substitution observed in the evolved genomes was significant (*p* = 0.003).

### Datasets Used for Bioinformatic Analysis

To test whether the extent of evolutionary compensation is influenced by the disrupted gene's pleiotropy, we used three complementary measures of gene pleiotropy. Environmental pleiotropy of a non-essential gene was defined as the number of unique conditions in which the removal of the gene resulted in a fitness defect according to Dudley and colleagues [27]. Network pleiotropy was measured as the total number of protein-protein interactions reported in the BioGRID database [57]. Finally, multifunctionality of a gene was calculated on the basis of a set of GO terms considered to be specific by yeast geneticists, as previously described [58].

To investigate whether mutations accumulated during compensatory evolution preferentially affected genes that are functionally related to the disrupted gene, we used different measures of functional relatedness: co-membership within stable protein complexes, shared functional category, genetic interaction profile similarity, co-expression, and paralogy. For protein complexes we used the manually curated dataset based on tandem affinity purification/mass spectrometry studies (YHTP2008) from the Wodak lab [59]. For functional categories, the MIPS Functional Catalogue Database was downloaded [60]. Genetic interaction profile similarities were obtained from a large-scale genetic interaction screen study [21]. The authors calculated the genetic interaction profile for a given gene deletion genotype as the list of genetic interaction scores detected across all other genes in their dataset. The genetic interaction profile similarity between two genes was defined as the Pearson correlation value of the two genetic interaction profiles [21]. For calculating co-expression data, 247 normalized microarray datasets from the M3D database [61] were used to create an expression profile for each gene. In case of multiple replicates per experiment, the average normalized values were calculated, and employed further. For each gene pair, co-expression value was calculated as the Pearson correlation coefficient between the two expression profiles.

Paralog gene pairs were identified by performing all-against-all BLASTP similarity searches of yeast open reading frames. We defined two genes as paralogs if (i) the BLAST score had an expected value E<10^−8^, (ii) alignment length exceeded 100 residues, (iii) sequence similarity was >30%, and (iv) they were not parts of transposons.

### Gene Expression Analysis

Eight evolved lines were selected for microarray analysis, all of them showing high fitness following evolution (at least 20% initial fitness defect compared to the wild-type control and at least 20% fitness improvement as a result of the evolutionary process). The corresponding ancestral strains and the wild-type control were also subjected to gene expression profiling. Table S3 contains the list of strains. Candidates were re-streaked and single clones were isolated and their fitness increase was confirmed by growth curve recording.

Two independent colonies of the wild-type control, evolved, and corresponding ancestor knock-out strains were inoculated into 15 ml YPD and grown overnight at 30°C. The saturated populations were diluted to an OD_600_ of 0.15 in 60 ml YPD and grown to early mid-log phase (OD_600_ 0.6±0.05) in 250 ml Erlenmeyer flasks with 220 rpm shaking at 30°C. Cells were harvested by centrifugation (4,000 rpm, 3 min, 30°C) and immediately frozen in liquid nitrogen after removal of supernatant. Total RNA was prepared by hot acidic phenol extraction and cleaned up using the QIAGEN's RNAeasy kit.

All steps after RNA isolation were automated using robotic liquid handlers as described previously [62]. Dual-channel 70-mer oligonucleotide arrays were used with a common reference pool of wild-type RNA. Quality control, normalization, and dye-bias correction was performed as described earlier [62]. The reported fold change is the average of the four replicate mutant profiles versus the average of all wild-type controls. A total of 58 transcripts showed stochastic changes in wild-type profiles and were excluded from the analyses. Differentially expressed genes were defined as those showing a 1.7-fold abundance change and a *p*-value<0.05 when comparing two strains. The raw dataset is available online at ArrayExpress (http://www.ebi.ac.uk/arrayexpress/, accession number E-MTAB-2352).

### Robustness of Results of the Transcriptome Analysis to Growth Rate Related Genes and Copy Number Variations

All transcriptome comparisons of the wild-type, knockout, and evolved strains were repeated on a dataset where CNVs, genes showing expression response to aneuploidy, and growth rate related genes were excluded. CNVs were identified on the basis of the read coverage of the genome sequence data (Table S2) with the exception of one strain (Δ*rpl43a*), which was not sequenced. In the case of Δ*rpl43a*, whole chromosome duplication was predicted on the basis of visual inspection of expression profiles. The position of partial chromosome duplication was predicted by the Charm algorithm [63]. In evolved strains carrying aneuploid chromosomes, genes showing expression response to that particular aneuploidy were excluded from the transcriptome comparisons (data on the transcriptome effects of aneuploidy were obtained from [64]). Genes showing significant expression response to changes in growth rate were also excluded, as defined previously [65] on the basis of the growth rate measurements of Brauer and colleagues [66].

### Strain Modifications to Investigate the Fitness Costs and Epistatic Effects of Compensatory Mutations

The evolved lines of Δ*mdm34* were chosen for in-depth genetic analysis. The fitness cost of the set of compensatory mutations accumulated in the evolved Δ*mdm34* lineages was measured in wild-type genetic background. To this end, the *MDM34* gene was re-introduced into the ancestor and evolved Δ*mdm34* lineages according to the *delitto perfetto* method [67]. First, the KanMX4 cassette in the ancestor and evolved Δ*mdm34* lineages was swapped with the CORE-UH cassette, containing the *KlURA3* and *hyg* markers. Then the *MDM34* open reading frame with longer than 0.3 kb flanking regions on both sides was amplified from the unevolved wild-type control strain and transformed into the cells to replace the CORE-UH cassette. The replacement of the *KlURA3* marker was counter-selected using 5-FOA containing medium. The loss of hyg^r^ was confirmed, the site and orientation of gene replacement was verified by PCR and the sequence of the *MDM34* gene was determined by capillary sequencing.

In a second analysis, a point mutation identified in the *MGA2* gene in one of the evolved Δ*mdm34* lineages was reinserted into both the wild-type and ancestor Δ*mdm34* background. This specific point mutation changes the 750th codon of *MGA2* from GAT to TAT resulting in the incorporation of tyrosine instead of aspartic acid. We refer to the mutant allele as *mga2-1*. Using the *delitto perfetto* method [67], we introduced this point mutation into the unevolved wild-type control strain. First, the CORE-UH cassette was inserted into the genome at the desired position of the SNP. Then, two complementary oligonucleotides of 81 bp length with the sequence of the region of interest and the SNP in the 41st position were transformed. The replacement of the *KlURA3* marker with the missense SNP was counter-selected using 5-FOA containing medium, loss of hyg^r^ was confirmed, and the result of the site-directed mutagenesis was verified by capillary sequencing. Attempts to introduce the *mga2-1* mutation into the ancestor Δ*mdm34* strain in this way were not successful, presumably due to the severe slow growth of the intermediate strain that lacks both *MDM34* and *MGA2* gene in a functional form. To complement this, a helper plasmid with *MDM34* gene (MoBY ORF Library [68]) was transformed into the cells prior to the site directed mutagenesis [69]. Because of the presence of the *URA3* marker on the helper plasmid, the CORE-Hp53 cassette was used in this experiment. The steps of mutagenesis were similar as without the helper plasmid, which was removed by passaging cells through 5-FOA afterwards.

### qPCR Method

Yeast samples were grown in 20 ml YPD medium to mid-log phase (0.8 OD600 value). RNA was extracted from 10^7^ yeast cells by acidic phenol method using TRI Reagent Protocol (Sigma-Aldrich Co). The RNA samples were concentrated by the NucleoSpin RNA Plant Kit (Macherey-Nagel), according to the manufacturer's instructions. A total of 500 ng RNA was used as a template to prepare cDNA using the Maxima First Strand cDNA Synthesis kit (Thermo Scientific). Reactions without template were set up to detect contaminations of the reagents used in the cDNA synthesis. qPCR reactions were set up in 20 µl volume, using the following templates: no template control, 10 ng non-transcribed RNA and cDNA transcribed from 10 ng RNA. The qPCR reactions were run in a Bioer LineK Gene device, using 2× Maxima SYBR Green qPCR Master Mix (Thermo Scientific). All samples had three technical replicates. Gene expression was determined in arbitrary units using a standard curve fitted on triplicates of a four-step 10-fold dilution series. *OLE1* expression level was determined relative to *TUB1* expression level as an internal control. All control reactions, not treated with reverse transcriptase or not having template, gave Ct values at least 10 cycles higher than the corresponding samples.

## Supporting Information

Figure S1 **Fitness trajectories often show a saturating trend by day 104 of the evolution experiment.** Fitness was measured at five time points during laboratory evolution (at day 0, 26, 52, 78, and 104), and fitness improvements were tested for each line and time interval (Wilcoxon rank-sum test, with a *p*-value cut-off of 0.05, see Methods and Table S10). (A) focuses on lines that showed one significant fitness improvement during the four 26-day time intervals. There is a strong (5-fold) depletion of lines that showed a fitness improvement in the last time step of the evolutionary experiment (eight out 159 cases, 40 expected, Chi-square test, *p*<10^−8^), indicating saturating compensatory evolution. (B) Representative examples of fitness trajectories showing a saturating trend (replicate lines of six genotypes are depicted).
(TIF)

Figure S2 **The extent of compensatory evolution in knock-outs is genotype-specific.** Here, we tested whether there are inherent differences in the propensity for compensation across genotypes (i.e., lines carrying different gene deletions). We defined compensatory evolution as a fitness increase that is disproportionally large relative to that in the evolving wild-type lines (Table S1). Accordingly, genotypes can be classified into three major categories on the basis of the fraction of corresponding lines fulfilling the above criteria (none, mixed, all). To assess the degree of departure from random expectation a randomization protocol was used. It generated a distribution of the above three categories under the assumption that all genotypes are equally likely to gain high fitness during the course of laboratory evolution. Specifically, the matrix of lines was shuffled one thousand times (gray bars) and the above categories were recalculated. The analysis revealed a strong enrichment of genotypes where all lines were compensated (“all”) and genotypes where none of the lines were compensated (“none”), while the “mixed” category was relatively rare (a). This result is not simply due to the fact that null mutations with more severe defects are especially likely to be compensated for. When only genotypes with similar initial fitness defects were considered, the trend remained (b,c,d). The four plots show the observed and randomly expected distributions a, for the whole dataset; b, c, d, for initial fitness ranges <0.7, 0.7–0.8, >0.8, respectively. Genotypes where either all or none of the evolutionary lines showed compensation are significantly enriched in all four cases, the corresponding Chi-square test *p*-values for a, b, c, and d are <10^−20^, 0.013, 7×10^−6^ and 10^−8^, respectively.
(TIF)

Figure S3 **Global transcriptome changes following compensatory evolution.** (A and B) were prepared by reproducing the main results of Figure 4, after excluding genes from the transcriptome profiles that (i) show copy number changes in the evolved lines, (ii) change expression level in aneuploid lines [13], or (iii) whose expression level depends on cellular growth rate (for details see Materials and Method). (A) The Euclidean distances of microarray profiles of the evolved evolutionary line from its ancestor and from wild type (WT) were calculated and normalized to the ancestor–wild type distance for each genotype (Table S11). The distances of the points on the figure are proportional to the calculated profile distances. For each genotype triplet, distances were calculated on the basis of those genes that are differentially expressed in at least one of the pairwise comparisons. (B) The figure focuses on the subset of genes that showed expression change upon gene deletion, and shows the fraction of these genes that changed expression during evolution in the opposite direction (i.e., evolution towards restoration of wild-type expression level; see inset). With one major exception (*Δmdm34*), only a small fraction of the expression changes were restored in the evolved lines (Table S11). The threshold for expression change was 1.7-fold-change and *p*<0.05, as previously described [14].
(TIF)

Figure S4 **Pleiotropic effects and mechanism of compensation of** ***Δmdm34*** **.** (A) Diversity of pleiotropic effects in independently evolved lines. Relative fitness across environments of isolated clones of independently evolving lines founded from the same *Δmdm34* genotype were measured as colony sizes grown on different media (Table S12). Genotypes are indicated on the left, the growth media are indicated above the heat map. For media composition and abbreviations, see Table S4. Values were normalized to that of the ancestral *Δmdm34* strain in the corresponding environment. In (A) and (D) log2 values are shown according to the color coding. (B) Quantitative PCR confirmation of upregulation of *OLE1* in both the evolved line carrying the *mga2-1* mutation and in the *Δmdm34 mga2-1* double mutant strain (Table S12). *OLE1* expression was measured relative to *TUB1* as an internal control and expression values were normalized to *Δmdm34* ancestor. Error bars show standard error. (C) Addition of oleic acid to the medium suppresses the fitness defect of *Δmdm34*, but does not affect the fitness of the evolved line carrying the *mga2-1* mutation or the strain carrying both *Δmdm34* and *mga2-1* mutations. Fitness was measured as colony sizes relative to unevolved wild-type control on rich media supplemented with DMSO as solvent control (non-treated), 0.1 mM oleic acid and 0.1 mM stearic acid (Table S12). For each genotype relative fitness change compared to the corresponding non-treated strain is shown. Error bars show standard error. (D) A specific point mutation in *MGA2* recapitulates the pleiotropic effects of compensatory evolution observed in evolved line 1. Relative fitnesses of *Δmdm34* evolving line 1, and *Δmdm34 mga2-1* double mutant were measured as colony sizes grown on different media (Table S12). Genotypes are indicated on the left, the growth media are indicated above the heat map. For media composition and abbreviations, see Table S4. Values were normalized to that of the ancestral *Δmdm34* strain in the corresponding environment.
(TIF)

Figure S5 **Validation of the phenotypic profiling experiment.** We compared our colony size measurements (Table S1) of the ancestral knockout strains to a published fitness profiling of the yeast deletion collection [4]. In the environments that match the published study, we find a good agreement between our data and the classification of Dudley and colleagues [4]. In each environment, knockouts present in our dataset were labeled as “no defect” versus “no/slow growth” based on Dudley and colleague's data. A significant difference was found between the two groups in our continuous fitness measurement (y-axis) in each of the environments (one-tailed Wilcoxon rank-sum test; */**/*** indicates *p*-value<0.05/0.01/0.001, respectively).
(TIF)

Table S1 **Fitness of strains in various environments.** The table includes fitness values of ancestor and evolved strains as measured in liquid YPD and in different agar media. Pleiotropy measures and GO process terms of the deleted genes are also presented.
(XLSX)

Table S2 **Mutations identified by Illumina next generation sequencing.** The table contains all the identified de novo and ancestral mutations in the sequenced genomes.
(XLS)

Table S3 **Microarray analysis results.** The table contains microarray data on all ancestral and evolved lines subjected to microarray analysis.
(XLS)

Table S4 **Composition of media used for phenotypic profiling.** Each media contained 1% yeast extract, 2% pepton, 2% agar, and different carbon sources. Some media also contained growth inhibitors as indicated. Concentration of drug inhibitors were set to have a minor but detectable growth inhibitory effect on the evolving wild-type control (unpublished data). The list of 14 growth media was primarily based on a previous study [4].
(XLSX)

Table S5 **Data supporting** **Figure 3D** **.** The table contains fitness measurements supporting dosage compensation of Δrpl6b by increased copy number of RPL6A.
(XLSX)

Table S6 **Data supporting** **Figure 4B and 4C** **.** (4B) Euclidean distances between pairs of wild-type evolved and ancestor knock-out strains, and also between the corresponding biological replicates. (4C) Categories of expression changes for each gene in the eight evolved knockout strains. Genes, which show initial expression change in the knockout can be categorized as restored, if expression during evolution goes in the opposite direction or unrestored if not. The category “other” includes genes not showing initial expression change.
(XLSX)

Table S7 **Data supporting** **Figure 6** **.** The table contains fitness (colony size) data employed for epistasis analysis between the mdm34 gene deletion and the mutations accumulated in the evolving strains (Figure 6B) and between the mdm34 gene deletion and one particular compensatory mutation (“mga2-1”) (Figure 6C) in both YPD and acetic acid, respectively.
(XLSX)

Table S8 **Data supporting** **Figure 7** **.** The table contains colony size measurement data on the environment-dependent compensation of the deletion of rpb9 by a loss-of-function mutation of whi2.
(XLSX)

Table S9 **Data supporting** **Figure 8** **.** Table includes single-gene knockout fitness and relative frequency of suppressing mutations for 3880 non-essential yeast genes.
(XLSX)

Table S10 **Data supporting Figure S1.** The table contains data on the fitness trajectories of the evolving strains. Fitness was measured at day 0, 26, 52, 78, and 104. The columns “improved day x-y” show whether there is a statistically significant fitness improvement between day x and y, as assessed by one-sided Wilcoxon tests (with false discovery rate correction, *p*<0.05 cutoff).
(XLSX)

Table S11 **Data supporting Figure S3.** (S3A) Euclidean distances between pairs of wild-type, evolved, and ancestor knock-outs, after excluding genes from the transcriptome profiles that (i) show copy number changes in the evolved lines, (ii) change expression level in aneuploid lines, or (iii) whose expression level depends on cellular growth rate. (S3B) Table includes categories of expression changes for each gene in the eight evolved knockout strains, excluding genes from the transcriptome profiles that (i) show copy number changes in the evolved lines, (ii) change expression level in aneuploid lines, or (iii) whose expression level depends on cellular growth rate. Genes displaying an initial expression change in the knockout can be categorized as restored, if its expression level changes in the opposite direction during evolution, or unrestored. The category “other” includes genes that did not display an initial expression change.
(XLSX)

Table S12 **Data supporting Figure S4.** The table contains data on the pleiotropic effects and mechanism of compensation of the deletion strain Δmdm34.
(XLSX)

Text S1 **Additional analyses supporting the prevalence of and mechanisms underlying compensatory evolution following gene loss.** The text includes a bioinformatic analyses of deleterious loss-of-function variants in natural yeast populations, a case study on compensatory mutations, and a brief description of image analysis used for measuring the extent of compensatory evolution.
(DOC)
