# Supplementary figures and images for: The Genomic Landscape of Compensatory Evolution
Source: PLoS Biol. 2014 Aug 26;12(8):e1001935. doi: 10.1371/journal.pbio.1001935 (PMC4144845; doi:10.1371/journal.pbio.1001935)

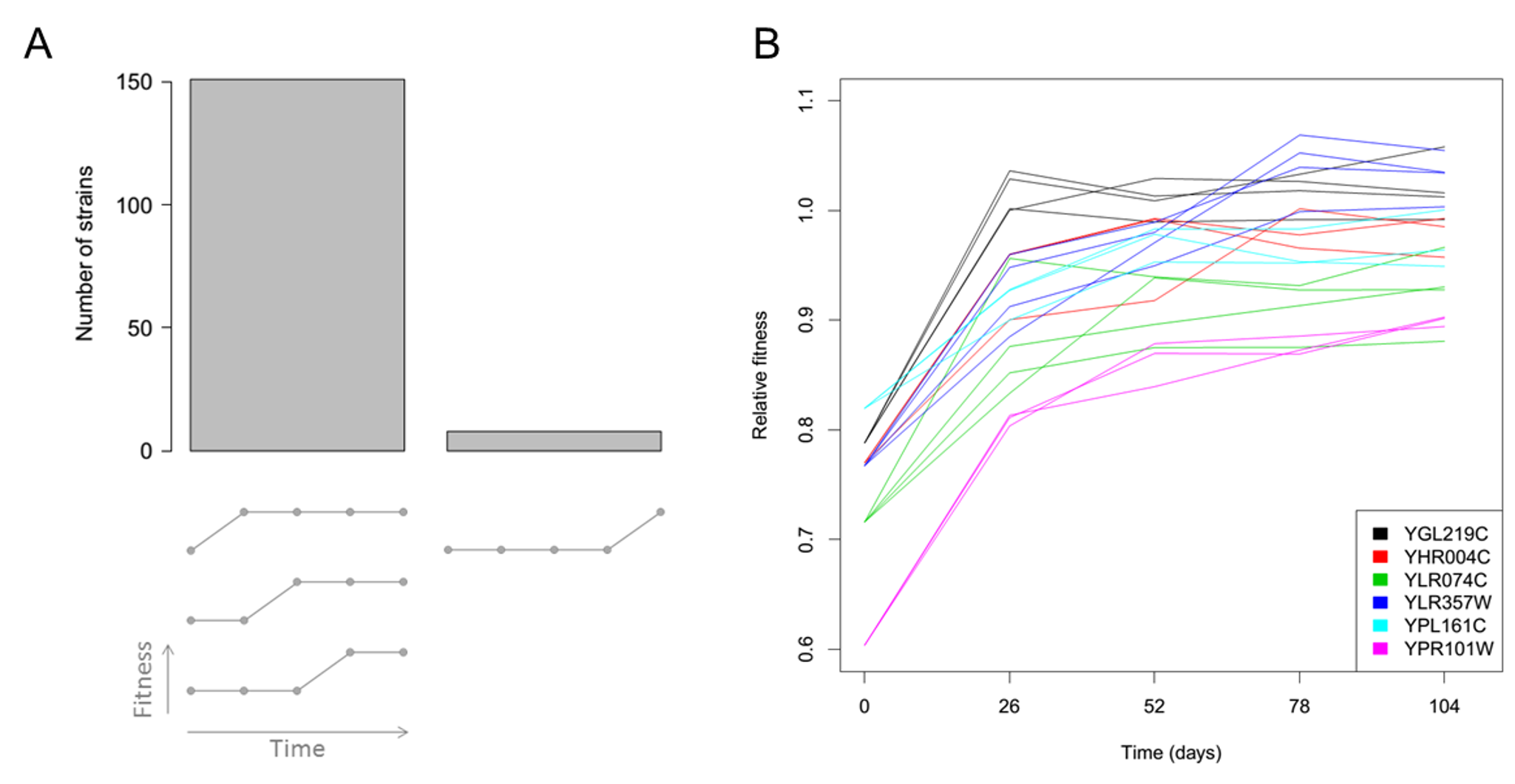

Supplement: Figure S1 — Fitness trajectories often show a saturating trend by day 104 of the evolution experiment. Fitness was measured at five time points during laboratory evolution (at day 0, 26, 52, 78, and 104), and fitness improvements were tested for each line and time interval (Wilcoxon rank-sum test, with a p-value cut-off of 0.05, see Methods and Table S10). (A) focuses on lines that showed one significant fitness improvement during the four 26-day time intervals. There is a strong (5-fold) depletion of lines that showed a fitness improvement in the last time step of the evolutionary experiment (eight out 159 cases, 40 expected, Chi-square test, p<10−8), indicating saturating compensatory evolution. (B) Representative examples of fitness trajectories showing a saturating trend (replicate lines of six genotypes are depicted). (TIF) [file pbio.1001935.s001.tif]

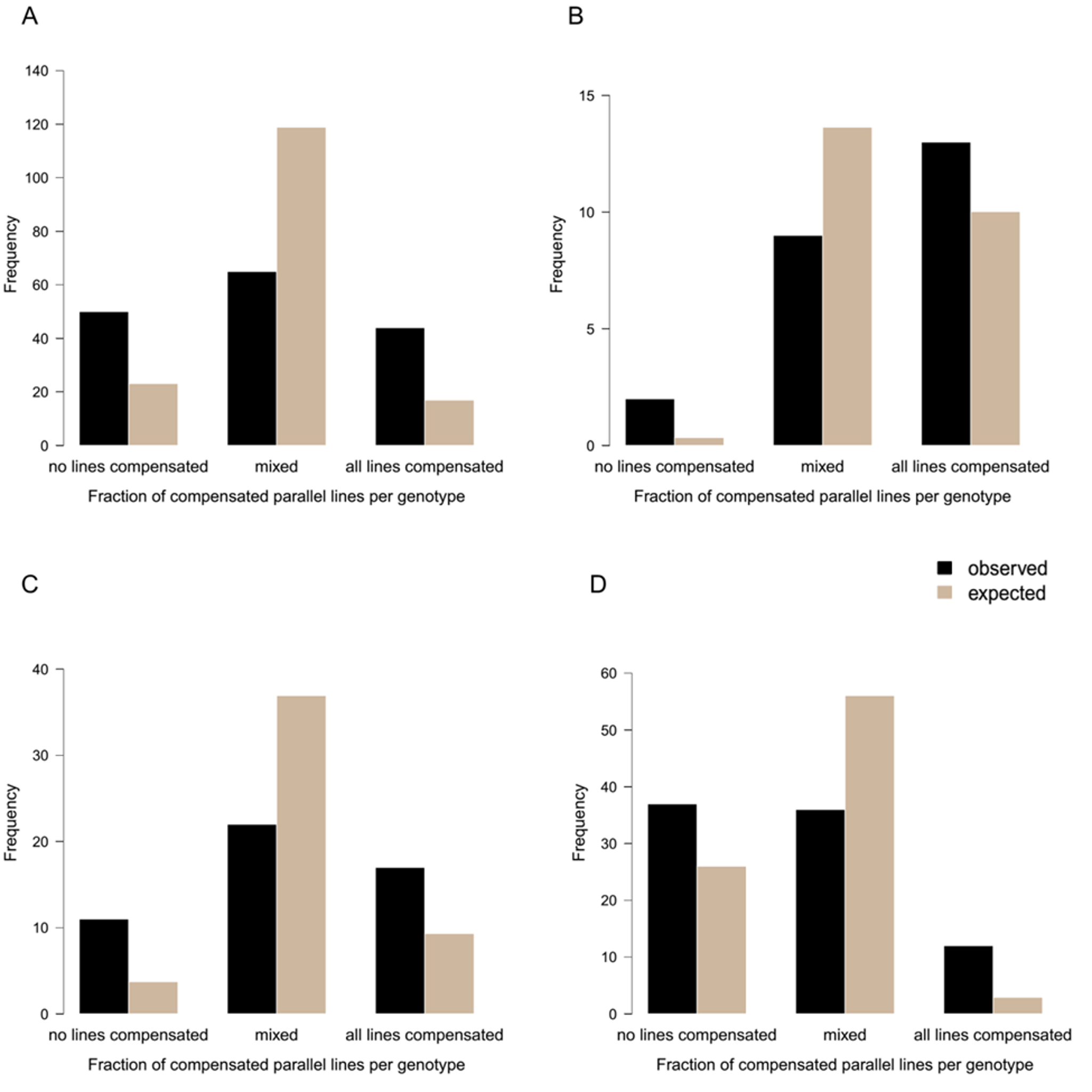

Supplement: Figure S2 — The extent of compensatory evolution in knock-outs is genotype-specific. Here, we tested whether there are inherent differences in the propensity for compensation across genotypes (i.e., lines carrying different gene deletions). We defined compensatory evolution as a fitness increase that is disproportionally large relative to that in the evolving wild-type lines (Table S1). Accordingly, genotypes can be classified into three major categories on the basis of the fraction of corresponding lines fulfilling the above criteria (none, mixed, all). To assess the degree of departure from random expectation a randomization protocol was used. It generated a distribution of the above three categories under the assumption that all genotypes are equally likely to gain high fitness during the course of laboratory evolution. Specifically, the matrix of lines was shuffled one thousand times (gray bars) and the above categories were recalculated. The analysis revealed a strong enrichment of genotypes where all lines were compensated (“all”) and genotypes where none of the lines were compensated (“none”), while the “mixed” category was relatively rare (a). This result is not simply due to the fact that null mutations with more severe defects are especially likely to be compensated for. When only genotypes with similar initial fitness defects were considered, the trend remained (b,c,d). The four plots show the observed and randomly expected distributions a, for the whole dataset; b, c, d, for initial fitness ranges <0.7, 0.7–0.8, >0.8, respectively. Genotypes where either all or none of the evolutionary lines showed compensation are significantly enriched in all four cases, the corresponding Chi-square test p-values for a, b, c, and d are <10−20, 0.013, 7×10^−6^ and 10−8, respectively. (TIF) [file pbio.1001935.s002.tif]

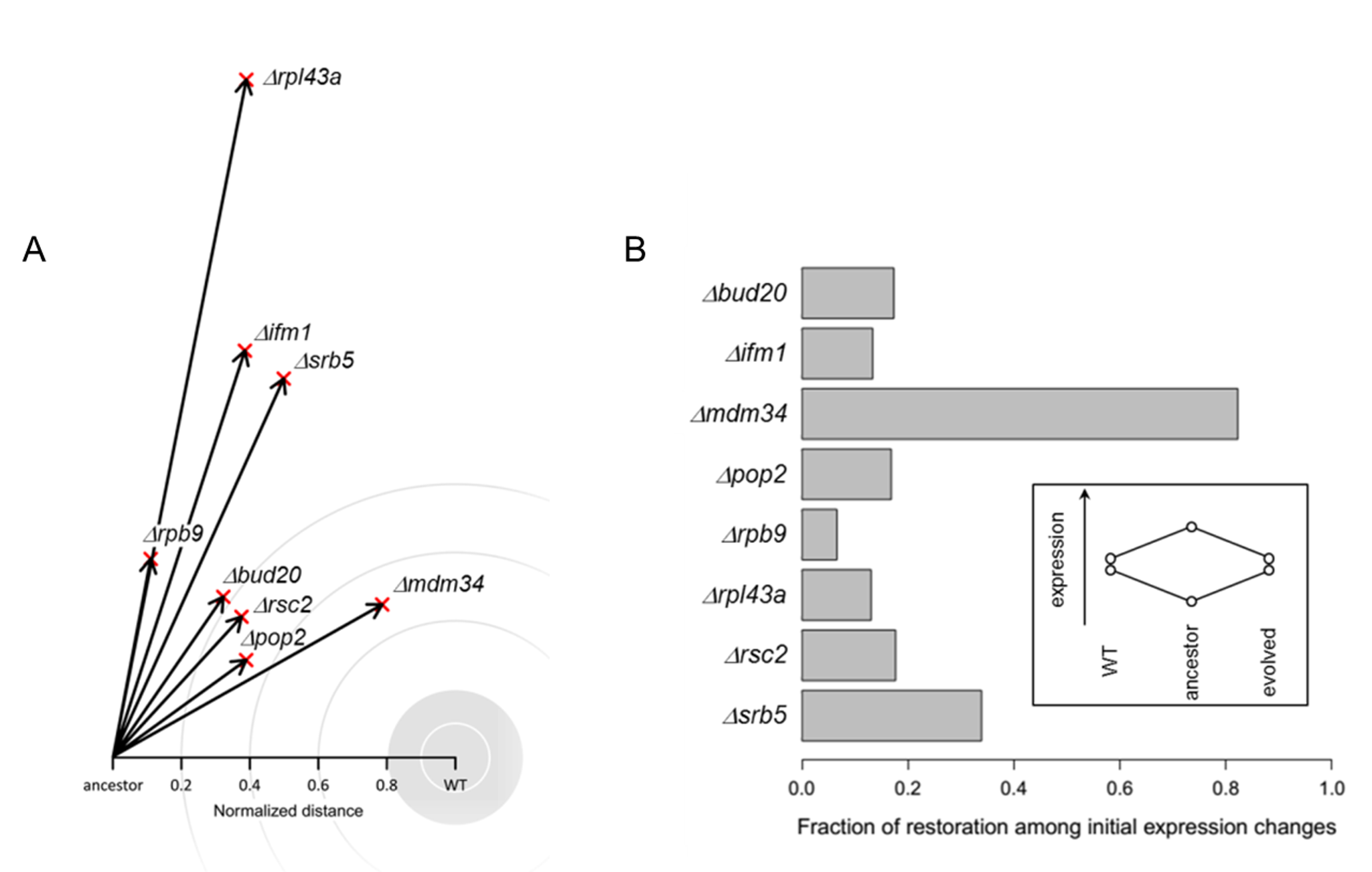

Supplement: Figure S3 — Global transcriptome changes following compensatory evolution. (A and B) were prepared by reproducing the main results of Figure 4, after excluding genes from the transcriptome profiles that (i) show copy number changes in the evolved lines, (ii) change expression level in aneuploid lines [13], or (iii) whose expression level depends on cellular growth rate (for details see Materials and Method). (A) The Euclidean distances of microarray profiles of the evolved evolutionary line from its ancestor and from wild type (WT) were calculated and normalized to the ancestor–wild type distance for each genotype (Table S11). The distances of the points on the figure are proportional to the calculated profile distances. For each genotype triplet, distances were calculated on the basis of those genes that are differentially expressed in at least one of the pairwise comparisons. (B) The figure focuses on the subset of genes that showed expression change upon gene deletion, and shows the fraction of these genes that changed expression during evolution in the opposite direction (i.e., evolution towards restoration of wild-type expression level; see inset). With one major exception (Δmdm34), only a small fraction of the expression changes were restored in the evolved lines (Table S11). The threshold for expression change was 1.7-fold-change and p<0.05, as previously described [14]. (TIF) [file pbio.1001935.s003.tif]

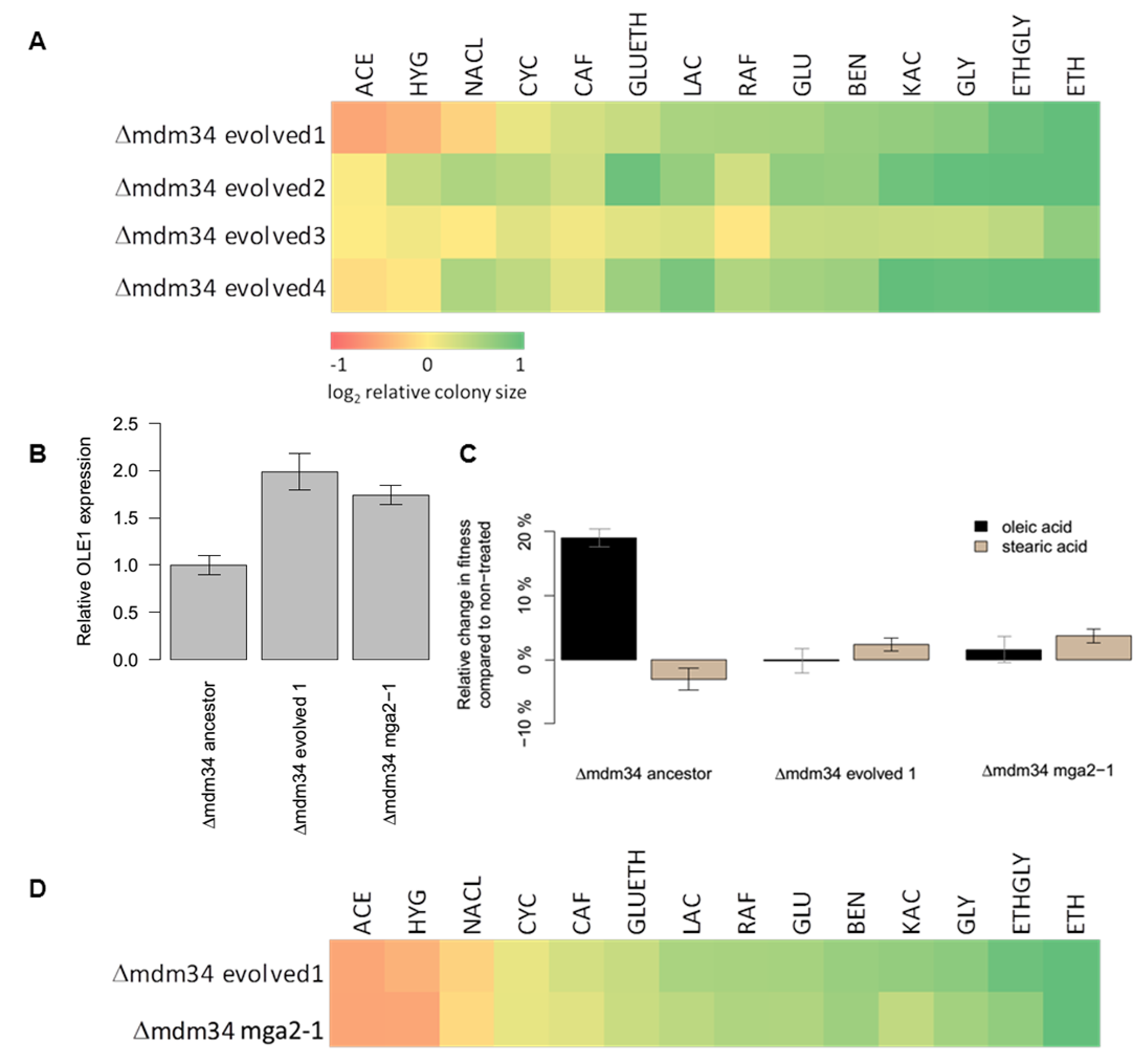

Supplement: Figure S4 — Pleiotropic effects and mechanism of compensation of Δmdm34 . (A) Diversity of pleiotropic effects in independently evolved lines. Relative fitness across environments of isolated clones of independently evolving lines founded from the same Δmdm34 genotype were measured as colony sizes grown on different media (Table S12). Genotypes are indicated on the left, the growth media are indicated above the heat map. For media composition and abbreviations, see Table S4. Values were normalized to that of the ancestral Δmdm34 strain in the corresponding environment. In (A) and (D) log2 values are shown according to the color coding. (B) Quantitative PCR confirmation of upregulation of OLE1 in both the evolved line carrying the mga2-1 mutation and in the Δmdm34 mga2-1 double mutant strain (Table S12). OLE1 expression was measured relative to TUB1 as an internal control and expression values were normalized to Δmdm34 ancestor. Error bars show standard error. (C) Addition of oleic acid to the medium suppresses the fitness defect of Δmdm34, but does not affect the fitness of the evolved line carrying the mga2-1 mutation or the strain carrying both Δmdm34 and mga2-1 mutations. Fitness was measured as colony sizes relative to unevolved wild-type control on rich media supplemented with DMSO as solvent control (non-treated), 0.1 mM oleic acid and 0.1 mM stearic acid (Table S12). For each genotype relative fitness change compared to the corresponding non-treated strain is shown. Error bars show standard error. (D) A specific point mutation in MGA2 recapitulates the pleiotropic effects of compensatory evolution observed in evolved line 1. Relative fitnesses of Δmdm34 evolving line 1, and Δmdm34 mga2-1 double mutant were measured as colony sizes grown on different media (Table S12). Genotypes are indicated on the left, the growth media are indicated above the heat map. For media composition and abbreviations, see Table S4. Values were normalized to that of the ancestral Δmdm34 strain i [file pbio.1001935.s004.tif]

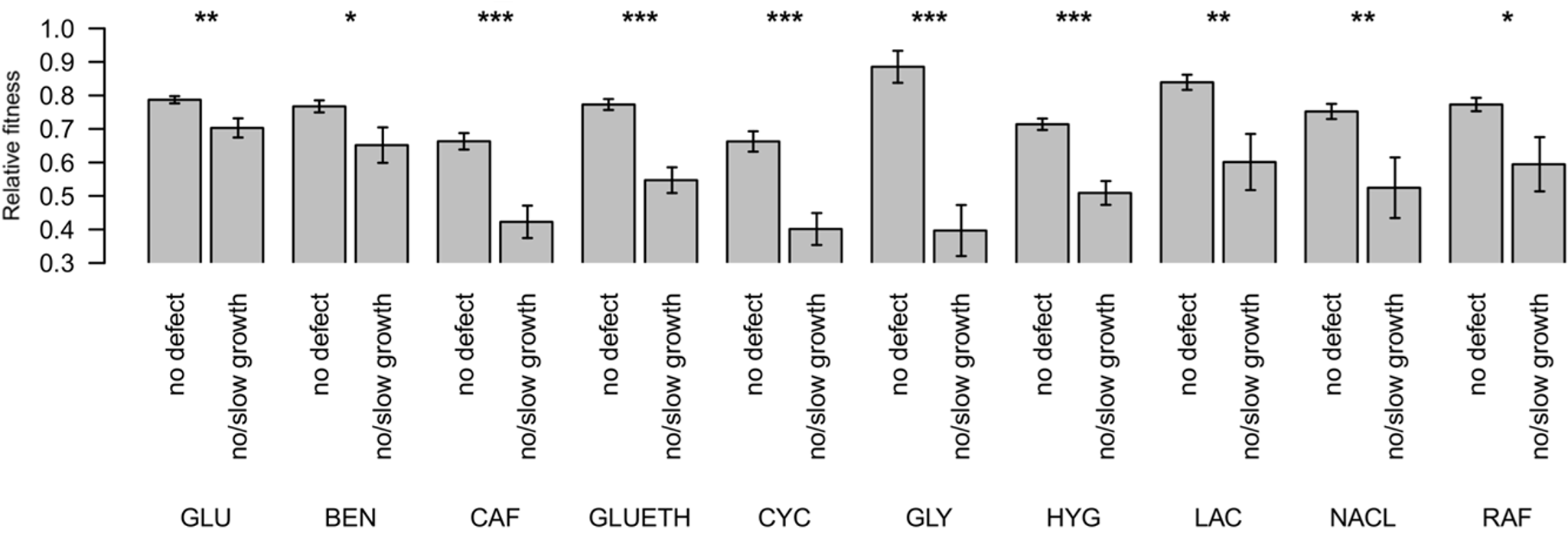

Supplement: Figure S5 — Validation of the phenotypic profiling experiment. We compared our colony size measurements (Table S1) of the ancestral knockout strains to a published fitness profiling of the yeast deletion collection [4]. In the environments that match the published study, we find a good agreement between our data and the classification of Dudley and colleagues [4]. In each environment, knockouts present in our dataset were labeled as “no defect” versus “no/slow growth” based on Dudley and colleague's data. A significant difference was found between the two groups in our continuous fitness measurement (y-axis) in each of the environments (one-tailed Wilcoxon rank-sum test; */**/*** indicates p-value<0.05/0.01/0.001, respectively). (TIF) [file pbio.1001935.s005.tif]
