# Supplementary material for: The Genomic Landscape of Compensatory Evolution
Source: PLoS Biol. 2014 Aug 26;12(8):e1001935. doi: 10.1371/journal.pbio.1001935 (PMC4144845; doi:10.1371/journal.pbio.1001935)
Supplement: Text S1 — Additional analyses supporting the prevalence of and mechanisms underlying compensatory evolution following gene loss. The text includes a bioinformatic analyses of deleterious loss-of-function variants in natural yeast populations, a case study on compensatory mutations, and a brief description of image analysis used for measuring the extent of compensatory evolution. (DOC) [file pbio.1001935.s018.doc]

Supporting Text S1

Deleterious loss-of-function variants are prevalent in yeast populations

Doniger and colleagues[1] sequenced the genome of two natural yeast strains and compared them to a reference genome (laboratory strain S288C) with the aim to identify deleterious and neutral DNA sequence variation within Saccharomyces cerevisiae. The paper found substantial evidence for functional variation in gene content and structure resulting from large insertions/deletions, frame-shifts and polymorphic start and stop codons. We analyzed this dataset further, and found that many of the identified loss-of function mutations would have serious fitness consequences in the reference (haploid) genome. First, we focused on large gaps in the genome alignments of the three strains (>5KB) most likely indicating insertion/deletion events across populations. Altogether, such gaps covered 211 genes (all of which are intact in the reference genome). Comparison of this dataset to results of previous genome-scale single gene knock-out screens [2-4] revealed that 12% of these 211 genes significantly reduce fitness in at least one laboratory condition when deleted. Similar results hold for point mutations which cause frame-shift in protein coding sequence regions. Out of the 379 genes affected by such events [1], 22% reduce fitness when deleted. These relatively high figures indicate that deleterious loss of function variants can reach high frequencies in yeast populations, which may be subjects of compensatory evolution.

Evolutionary compensation in lines carrying deletion in MDM34

MDM34 is a subunit of the endoplasmatic reticulum - mitochondria tethering complex (ERMES complex). Deletion of MDM34 reduces the levels of cardiolipin (CL) [5]. CL is a mitochondrium-specific phospholipid, and has a critical for the biogenesis of respiratory chain supercomplexes. It binds to cytochrome c, limiting its soluble pool [6]. Mitochondrial CL is mainly composed of mono-unsaturated and di-unsaturated fatty acyl chains of 16–18 carbons in length [6]. Despite of the substantial fitness defect associated to MDM34 deletion, the fitnesses of all four ∆mdm34 independently evolving lines were uniformly high and comparable to those of the wild type in the medium of laboratory evolution (Table S1). However, the fitnesses of these replicate lines showed a large variation across environments (Figure S4), indicating differences in the underlying genetic mechanisms of compensation. Consequently, these lines are especially suitable candidates for an in-depth analysis to understand the nature and molecular mechanisms of compensatory evolution.

Upregulation of unsaturated fatty acid synthesis (evolved line 1)

A point mutation in the MGA2 gene (referred to as mga2-1) was found in one of the evolved ∆mdm34 lines. MGA2 positively regulates the transcription of an essential fatty acid desaturase (OLE1). OLE1 is required for the production of monounsaturated fatty acids [7], which are precursors of cardiolipin [8]. Real-time PCR analysis demonstrated that the ∆mdm34 evolved line harboring the mga2-1 mutation showed two-fold enhancement in the expression of OLE1 compared to the expression level of this gene in the corresponding ancestor, (t-test, P<0.05, see Figure S4B).

We propose that upregulation of OLE1 transcription via mutation in MGA2 increases the production of unsaturated fatty acids, and as consequence, increases CL production in ∆mdm34 by mass action. According to this hypothesis, cell growth is limited by the availability of desaturated fatty acids in ∆mdm34 but not in the corresponding evolved line with mga2-1 mutation. Several lines of evidence support this hypothesis (see below).

Oleic acid is the main primary product of Ole1 protein, as addition of oleic acid complements OLE1 null mutation [7]. As a consequence, administration of oleic acid into the medium partially compensated the fitness defect of ∆mdm34 (Figure S4C). As expected, this effect was specific to the unsaturated oleic acid, since administration of the saturated stearic acid with the same carbon chain length did not provide compensation.

However, the external oleic acid failed to increase the fitness of the evolved line, indicating that cell growth is no longer limited by the availability of the desaturated fatty acid in the presence of an mga2-1 mutation (Figure S4C). To further support our hypothesis, we introduced the mga2-1 mutation into ∆mdm34. As expected, OLE1 was upregulated in this strain (t-test, p<0.05, see Figure S4B), and addition of external unsaturated fatty acid to the medium had no fitness effect (Figure S4C).

Environmental dependence of the phenotypic effects of mga2-1

As a next step, the fitness of the ∆mdm34 mga2-1 double mutant was measured in 14 environments. The fitness profile of the double mutant showed strong positive correlation with that of the corresponding evolved line (Spearman rho= 0.94 p<10^-6^, see Figure S4D), demonstrating that this double mutant captures the fitness trade-offs of evolved line 1. Remarkably, mga2-1 mutations failed to compensate loss of mdm34 in an acidic environment (Figure 6C). We suspect that this pattern is partly due to a dominant downregulation of OLE1 upon mild acidic stress [9].

Cardiolipin biosynthesis gene MDM35 (evolved line 3)

Mdm35p is a mitochondrial intermembrane space protein, and its physical association with Ups1p is required for the normal function of Ups1p in cardiolipin biosynthesis [10]. The identified mutation in MDM35 may facilitate the Ups1p dependent step of cardiolipin biosynthesis in the intermembrane space, downstream of the phospholipid transport, which is catalyzed by the ERMES complex.

Carnitine transporter (evolved line 4)

Crc1p is a mitochondrial inner membrane carnitine transporter. It is involved in the carnitine-dependent transport of acetyl-CoA from peroxisomes to mitochondria during fatty acid beta-oxidation [11]. Crc1p also transports long-chain acylcarnitines, albeit at lower efficiency [11], which may provide an alternative route for long chain unsaturated acyl groups to enter the cardiolipin synthesis pathway. Moreover, the free carnitine in the mitochondrion activates respiration through interaction with cardiolipin at the membrane level [12]. We speculate that the identified point mutation in the CRC1 gene acts through the modulation of the mitochondrial carnitine pool, and indirectly modulates cardiolipin functioning in respiration.

Computational pipeline of image analysis and colony growth normalization

Images of plates with colony arrays were photographed in a dark-room with a backlit plate mount. We have developed a JAVA software to process images and calculate colony sizes. First, grid positions on the plate were triangulated using reference spots on the plate mount. Then a threshold level was set manually on the grayscale histogram of a randomly chosen image of a particular experiment (the same threshold was used for all plates of one particular experiment). Then a binary image was obtained by thresholding and round objects were detected by Hough transformation. Objects were accepted as true colonies if they fit the following criteria: appropriate Hough value, complete separation from neighboring objects and circularity. All these parameters had been optimized to enable efficient distinction of true colonies from irrelevant objects such as bubbles, scratches and image noise. Finally, a multilayer image with the original image and the identified colonies was generated and manually confirmed for all analyzed plates. The number of pixels occupied by the identified colonies was used as a measure of fitness.

Colony growth in high-density arrays is often affected by various systematic effects [8]. Thus, we developed a Matlab script to identify and minimize these effects. First, colonies located in the two outermost rows and columns were excluded. Next, all colony sizes were normalized by the median size of isogenic wild type control distributed evenly across the same plate. Next, to identify potential systematic biases of colony size measurement, we calculated the error of measurement (eom) value for each colony using the following formula:

eom=(x-µ)/µ

where x is the size of the actual colony and µ is the mean of the colony sizes of the same genotype. Thus eom estimates the relative deviation of the colony size from the expected value of its genotype. The matrix of eom values was used to detect systematic biases. The detected plate-, row- and spatial-effects were then corrected in case of all colonies [8]. Both softwares are available upon request from the authors.

References

1 Doniger SW, Kim HS, Swain D, Corcuera D, Williams M, et al (2008) A catalog of neutral and deleterious polymorphism in yeast. PLoS genetics 4: e1000183.

2 Giaever G, Chu AM, Ni L, Connelly C, Riles L, et al (2002) Functional profiling of the Saccharomyces cerevisiae genome. Nature 418: 387-391.

3 Deutschbauer AM, Jaramillo DF, Proctor M, Kumm J, Hillenmeyer ME, et al (2005) Mechanisms of haploinsufficiency revealed by genome-wide profiling in yeast. Genetics 169: 1915-1925.

4 Dudley AM, Janse DM, Tanay A, Shamir R, Church GM (2005) A global view of pleiotropy and phenotypically derived gene function in yeast. Mol Syst Biol 1: 2005.0001.

5 Osman C, Haag M, Potting C, Rodenfels J, Dip PV, et al (2009) The genetic interactome of prohibitins: coordinated control of cardiolipin and phosphatidylethanolamine by conserved regulators in mitochondria. J Cell Biol 184: 583-596.

6 Joshi AS, Zhou J, Gohil VM, Chen S, Greenberg ML (2009) Cellular functions of cardiolipin in yeast. Biochim Biophys Acta 1793: 212-218.

7 Stukey JE, McDonough VM, Martin CE (1990) The OLE1 gene of Saccharomyces cerevisiae encodes the delta 9 fatty acid desaturase and can be functionally replaced by the rat stearoyl-CoA desaturase gene. J Biol Chem 265: 20144-20149.

8 Baryshnikova A, Costanzo M, Kim Y, Ding H, Koh J, et al (2010) Quantitative analysis of fitness and genetic interactions in yeast on a genome scale. Nat Methods 7: 1017-1024.

9 Mira NP, Teixeira MC, Sá-Correia I (2010) Adaptive response and tolerance to weak acids in Saccharomyces cerevisiae: a genome-wide view. OMICS 14: 525-540.

10 Connerth M, Tatsuta T, Haag M, Klecker T, Westermann B, Langer T (2012) Intramitochondrial transport of phosphatidic acid in yeast by a lipid transfer protein. Science 338: 815-818.

11 Palmieri L, Lasorsa FM, Iacobazzi V, Runswick MJ, Palmieri F, Walker JE (1999) Identification of the mitochondrial carnitine carrier in Saccharomyces cerevisiae. FEBS Lett 462: 472-476.

12 Battelli D, Bellei M, Arrigoni-Martelli E, Muscatello U, Bobyleva V (1992) Interaction of carnitine with mitochondrial cardiolipin. Biochim Biophys Acta 1117: 33-36.

13 Torres EM, Sokolsky T, Tucker CM, Chan LY, Boselli M, et al (2007) Effects of aneuploidy on cellular physiology and cell division in haploid yeast. Science 317: 916-924.

14 van Wageningen S, Kemmeren P, Lijnzaad P, Margaritis T, Benschop JJ, et al (2010) Functional overlap and regulatory links shape genetic interactions between signaling pathways. Cell 143: 991-1004.

15 Costanzo M, Baryshnikova A, Bellay J, Kim Y, Spear ED, et al (2010) The genetic landscape of a cell. Science 327: 425-431.
